# Natural Adeno-Associated Virus Serotypes and Engineered Adeno-Associated Virus Capsid Variants: Tropism Differences and Mechanistic Insights

**DOI:** 10.3390/v16030442

**Published:** 2024-03-12

**Authors:** Estrella Lopez-Gordo, Kyle Chamberlain, Jalish Mahmud Riyad, Erik Kohlbrenner, Thomas Weber

**Affiliations:** 1 Affinia Therapeutics, Waltham, MA 02453, USA; 2 Independent Researcher, South Plainfield, NJ 07080, USA; 3 Spark Therapeutics, Philadelphia, PA 19104, USA

**Keywords:** AAV receptor, AAV engineering, directed evolution, CNS, brain-blood barrier

## Abstract

Today, adeno-associated virus (AAV)-based vectors are arguably the most promising in vivo gene delivery vehicles for durable therapeutic gene expression. Advances in molecular engineering, high-throughput screening platforms, and computational techniques have resulted in a toolbox of capsid variants with enhanced performance over parental serotypes. Despite their considerable promise and emerging clinical success, there are still obstacles hindering their broader use, including limited transduction capabilities, tissue/cell type-specific tropism and penetration into tissues through anatomical barriers, off-target tissue biodistribution, intracellular degradation, immune recognition, and a lack of translatability from preclinical models to clinical settings. Here, we first describe the transduction mechanisms of natural AAV serotypes and explore the current understanding of the systemic and cellular hurdles to efficient transduction. We then outline progress in developing designer AAV capsid variants, highlighting the seminal discoveries of variants which can transduce the central nervous system upon systemic administration, and, to a lesser extent, discuss the targeting of the peripheral nervous system, eye, ear, lung, liver, heart, and skeletal muscle, emphasizing their tissue and cell specificity and translational promise. In particular, we dive deeper into the molecular mechanisms behind their enhanced properties, with a focus on their engagement with host cell receptors previously inaccessible to natural AAV serotypes. Finally, we summarize the main findings of our review and discuss future directions.

## 1. Introduction

Over the last two decades, the field of creating “designer” adeno-associated virus (AAV) vectors has nothing short of exploded, and a number of reviews discussing different aspects of these research efforts, often heavily focused on technologies for the discovery of novel AAV variants, have been published [1,2,3,4,5,6,7]. In contrast, the significance of tissue-specific receptor expression and, especially, the role of intracellular trafficking and processing of the capsid in tissue/cell-specific gene expression has not been discussed in detail. Here, we discuss not only the pros and cons of the various capsid engineering approaches but also the function of receptors and trafficking pathways on transduction.

Targeting the central nervous system (CNS) is a particular fertile ground for the discovery of novel AAV variants. Identifying AAV capsid variants able to cross the blood–brain barrier (BBB) is a challenging endeavor. However, the plethora of diseases that affect specific cell types or large areas of the brain, for instance, Alzheimer’s disease and prefrontal dementia, make the isolation of AAVs that can deploy their payload broadly to the CNS upon systemic delivery critical. Hence, a significant part of this review focuses on seminal discoveries in this area of research. However, the sheer number of publications in this area makes it impossible to review them all in detail; thus, we summarize further details and additional relevant variants in Supplementary Table S1.

To our knowledge, AAV capsid engineering technology and the role of receptors and intracellular trafficking on cell type and tissue specificity have not been discussed in this level of detail in one review before. We also believe that the comprehensive review of neurotropic capsids is of particular interest to both research neuroscientists and clinical neurologists. Taken together, we hope that our review will be a valuable resource for a broad swath of people working in the field of AAV gene therapy.

## 2. Discovery of AAV and Their Evolution as Gene Therapy Tools

**Figure 1:**
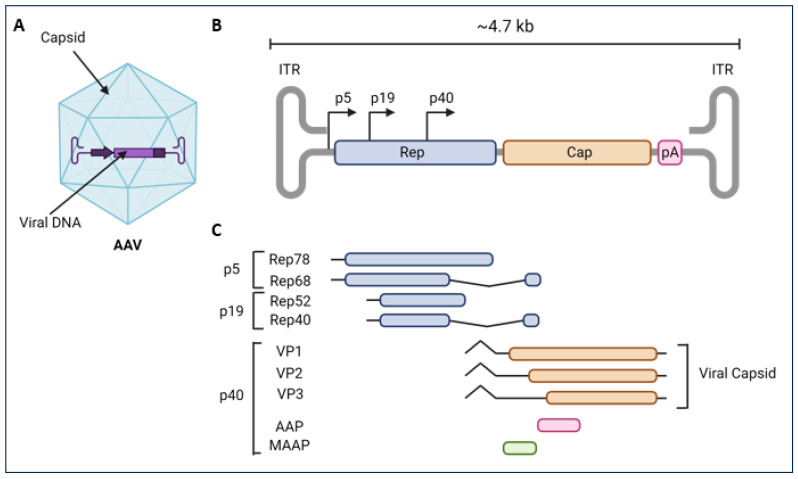
(**A**) Adeno-associated virus serotype (AAV) particle with the AAV single-stranded genome. (**B**) AAV2 genome organization including two inverted terminal repeats (ITs) flanking the *rep* and *cap* genes, *cap* polyA, and AAV promoters (p5, p19, and p40). (**C**) Transcriptional map of AAV structural (VP1, VP2, and VP3) and non-structural proteins (Rep78, Rep68, Rep52, Rep40, AAP, and MAAP). VP: viral protein; AAP: assembly-activating protein; and MAAP: membrane-associated AAV protein. Images created with Biorender (available at: https://www.biorender.com).

AAV was originally discovered as a contaminant of adenovirus preparations, and it was found to require the presence of adenovirus [8,9,10,11], HSV [12,13], vaccinia virus [14], or human papilloma virus [15] to replicate. Because of its dependence on other viruses, AAV became the founding member of the genus *Dependoparvovirus* within the *Parvoviridae* family. The wild-type AAV2 (wtAAV2) ssDNA genome is ~4.7 kb in length [16], with two 145 bp long inverted terminal repeats (ITR) which form T-shaped hairpin structures, work as the self-priming origins of replication [17] on the left hand and right side of the AAV2 the genome [18,19,20], and play an important role in integration and packaging [17,21] (Figure 1). The ITRs also flank the *rep* and *cap* genes. Within the *rep* region, three promoters (p5, p19, and p40) drive the transcription of six distinct mRNAs [22,23]. Transcription from the p5 promoter results in two large rep proteins through alternative splicing (Rep78 and Rep68). Similarly, the p19 promoter drives transcription of two smaller Rep proteins (Rep52 and Rep40). In the presence of a helper virus, the large rep proteins play a central role in replication, transcription control, and almost all other aspects of the viral life cycle. Strikingly, in the absence of a helper virus, the large rep proteins catalyze the insertion of the wtAAV2 genome into the human genome, preferentially into the AAVS1 region located on human chromosome 19 [24,25]. The smaller rep proteins (Rep52 and, especially, Rep40) are essential for genome packaging into preformed capsids [26].

The p40 promoter drives the expression of the two differentially spliced transcripts from the *cap* gene. Together, these transcripts encode three capsid protein subunits (VP1, VP2, and VP3) that make up the icosahedral AAV capsid [27] (Figure 1). The virion is made up of a total of 60 subunits of VP1, VP2, and VP3, with heterogenous and stochastic ratios which vary between 1:1:10 and 1:1:18, depending on the serotype, expression levels, and production system [27,28,29,30]. One of the two mRNA splice variants encodes VP1, and the other encodes VP2 and VP3 [31,32]. Importantly, the three capsid protein subunits share the C-terminal sequence region of VP3 [33,34]. The VP1/VP2 overlapping region (lacking in VP3) contains two basic regions (BR) [35] that work as a nuclear localization signal (NLS) during AAV infection [36]. The VP1 N-terminal unique region (VP1u) harbors yet another NLS as well as a phospholipase A2 (PLA2) domain that is critical during the infection process [37]. In addition, there is an alternative ORF within the *cap* gene coding for the assembly-activating protein (AAP), which shuttles newly synthesized capsid monomers into the nucleolus, thereby facilitating efficient capsid assembly, although it is not essential for all serotypes [38,39,40]. Recently, another ORF in the VP1/2 region of *cap* coding for a protein called membrane-associated accessory protein (MAAP) has been discovered [41]. MAAP localizes to the plasma membrane, perinuclear membrane structures, and nuclear membrane [42] and has been reported to play a role in infectivity [41], replication, and egress from infected cells [42,43].

**Figure 2:**
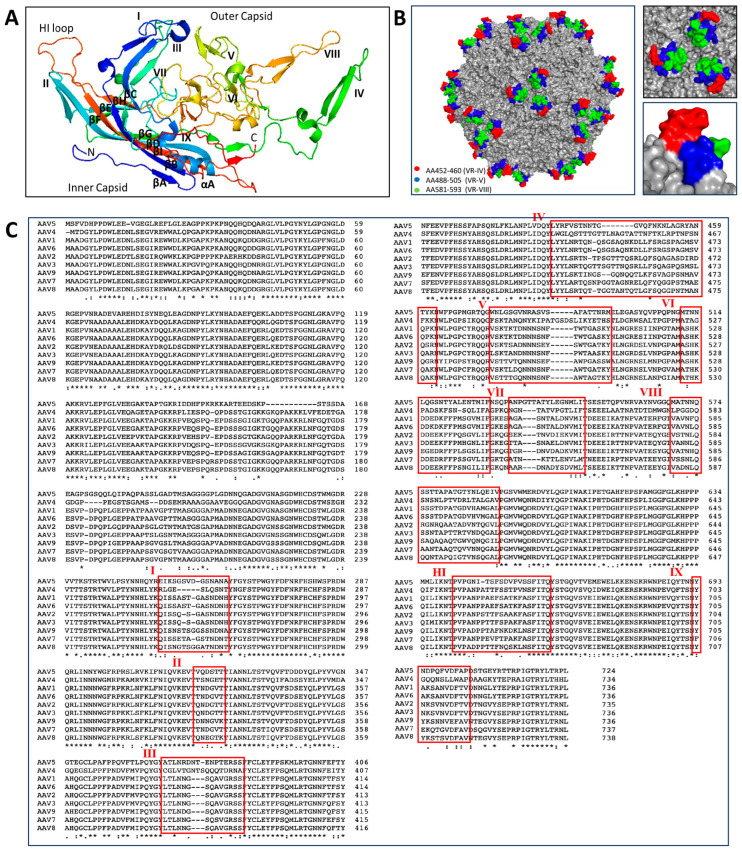
(**A**) Ribbon diagram of the AAV9 viral protein monomer colored by chain and with labelled β-sheet strands, α helix, VRs, and C/N-terminus. VR location within protein chains: VR-I (blue), VR-II (teal), VR-III (dark blue), VR-IV (green), VR-V and VR-VI (yellow), VR-VII (dark yellow), VR-VIII (orange), and VR-IX (red). (**B**) Adeno-associated virus (AAV) capsid surface model illustrates the location of variable regions (VR)-IV, V, and VIII (left panel). Representation of the AAV9 capsid zoomed-in (right panels), showing VR-IV at AA452-460 (red) and VR-VIII at AA581-593 (green) from a monomer and VR-V at AA488-505 (blue) from the adjacent monomer of the three-fold axis. (**C**) Comparison of VP1 amino acid sequences from AAV serotypes 1, 2, 3, 4, 6, 7, 8, and 9. VRs [43] are highlighted in red boxes. Multi-sequence alignment in (**C**) performed with Clustal Omega (available at: https://www.ebi.ac.uk/Tools/msa/clustalo/). Images in (**A**,**B**) constructed with PyMOL Molecular Graphics System Version 2.5.5, Schrodinger, LLC (Boston, MA, USA).

At least 13 natural AAV serotypes and more than 100 different variants have been isolated [44,45]. The existing serotypes share between 53 and 99% amino acid sequence homology in each structural protein subunit [46] but have different tissue tropism and use a diverse range of receptors and co-receptors for cellular entry [5,47]. Although X-ray crystallography and, more recently, cryo-electron microscopy (cryo-EM) of AAV capsids [48,49,50,51,52,53,54,55] revealed high similarities in capsid surface topology across serotypes, most of the differences in tissue tropism can be traced back to their capsid structures. Each AAV capsid subunit contains nine surface-exposed hypervariable regions (VRs), in which genome divergence across serotypes is most concentrated [49,53,54] (Figure 2A,C). Each subunit interfaces with other neighboring subunits through intricate protein–protein interactions defined by the two-, three-, and five-fold symmetry axis of the icosahedral AAV particle. Near the two-fold axis of symmetry, the most conserved surface features confer structural stability to the capsid. The five-fold axis harbors a cylindrical feature surrounded by a depression and is involved in the encapsidation of AAV genomes [56]. The three-fold axis is characterized by the three largest outer protrusions [57] and contains the most exposed VRs (VR-IV, VR-V, and VR-VIII), which are involved in receptor engagement, thus being key determinants of tropism [45,49,58,59] (Figure 2B).

Perhaps the most important finding for AAV gene therapy was that, to produce recombinant AAV (rAAV), as much as 96% of the wtAAV2 genome could be replaced with foreign sequences (e.g., genes of interest (GOIs) or therapeutic genes) [60], as long as the ITRs were retained for viral genome replication [61] and the other viral proteins were provided in trans. Importantly, rAAVs are capable of driving long-term transgene expression and induce a weak cellular immune response and a low vector-related toxicity in vivo, highlighting their great potential as safe and effective gene therapy vectors [62,63,64,65,66].

The first AAV clinical trial on humans was approved in 1995. In this trial, a patient with cystic fibrosis was infected with an rAAV2 vector encoding the cDNA of the *CFTR* gene [67]. Since then, around 350 clinical trials with AAV vectors have been initiated worldwide [68], and AAV has become arguably one of the most popular vectors for in vivo gene therapy, mainly for targeting eyes, liver, central nervous system, and muscle [69]. In 2012, GLYBERA^TM^, the first ever in vivo AAV gene therapy product (AAV1-based), was approved by the European Medicines Agency (EMA) to treat lipoprotein lipase (LPL) deficiency [70]. In 2017, the FDA approved LUXTURNA^TM^ (AAV2-based) for correcting Leber Congenital Amaurosis type 2 (LCA2) [71,72] and approved ZOLGENSMA^TM^ (AAV9-based) in 2019 to treat children affected by Spinal Muscular Atrophy (SMA) [73,74]. In 2022, EMA also approved UPSTAZA^®^ (AAV2-based) to treat Aromatic L-amino Acid DeCarboxylase (AADC) [75] and HEMGENIX^TM^ (AAV5-based) to treat hemophilia B in adult patients [76]. In 2023, ROCTAVIAN^TM^ (AAV5-based) received FDA approval to treat hemophilia A in adult males [77,78] and Elevidys (AAVrh74-based) to treat Duchenne muscular dystrophy (DMD) [79]. In addition to these products, a number of AAV-based therapeutics are showing promise in clinical trials in treating several other diseases [69].

## 3. Transduction Mechanisms of Natural AAV Serotypes

**Figure 3:**
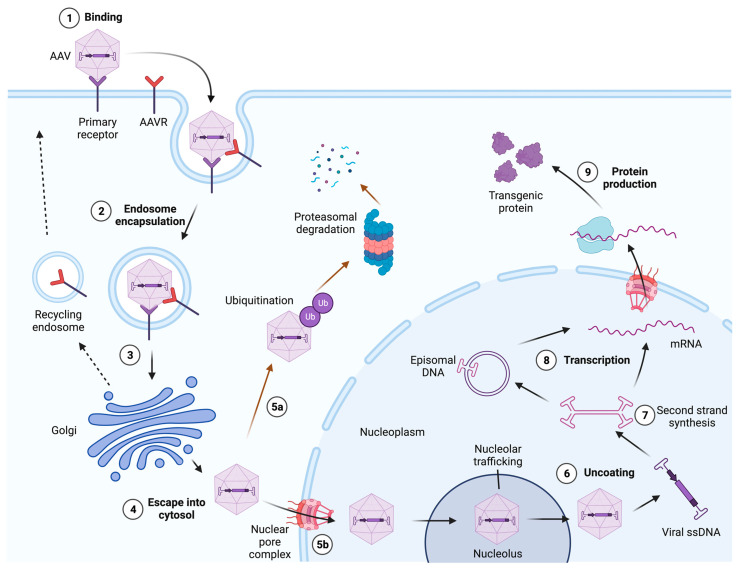
Model of entry and intracellular trafficking of adeno-associated virus (AAV) vectors. Following interactions with receptors and co-receptors and with the help of essential host cell components such as AAVR (1), AAV enters the target cell by means of endocytosis (2) and travels inside an endocytic vesicle toward the trans-Golgi network via retrograde trafficking (3). Acidification of the endosome and, potentially, additional factors trigger major structural changes within the capsid that expose a phospholipase A2 (PLA2) catalytic domain, allowing the virion to escape into the cytosol (4). The AAV virion can then be ubiquitinated for degradation (5a) or be imported into the nucleus through the nuclear pore complex (5b). In the nucleus, it accumulates in the nucleolus before moving into the nucleoplasm, where the single-stranded genome is released (6) and converted into a double-stranded DNA which can persist in the nucleus as a circular episome or as linear or episomal concatemers (7), leading to gene expression (8) and protein production (9). Image created with Biorender (available at https://www.biorender.com).

Much of our knowledge about AAV biology comes from studies with AAV2. Regardless of the different serotypes and their respective tropism, the transduction mechanisms appear to be similar and have been described in detail elsewhere [80,81] (Figure 3). Briefly, AAV enters the cell by endocytosis following receptor and/or co-receptor binding. Aided by essential host cell factors such as the AAV receptor (AAVR) [82,83], after having entered the cell, AAV starts retrograde trafficking towards the trans-Golgi network (TGN) inside an endocytic vesicle. During entry, endosomal acidification and, likely, additional factors cause major structural changes within the capsid that lead to the escape of the virion into the cytosol [84,85]. Next, the intact capsid enters the nucleus through the nuclear pore complex (NPC) [86], where the single-stranded genome is released from the capsid and converted into a double-stranded DNA (dsDNA) molecule. The dsDNA can then persist as a circular episome or as linear or episomal concatemers and express viral genes or recombinant transgenes. It is worth noting that, in rare cases, the recombinant AAV genome can also integrate into the host genome [87,88,89].

### 3.1. AAV Cell Surface Receptors

Receptors for most AAV serotypes have been identified (Table 1). Summerford et al. first established heparan sulfate proteoglycans (HSPG) as a primary receptor for AAV2 [90], and, later, two arginine residues (R585 and R588) and three more basic residues (R484, R487, and K532) were found to be critical for this interaction [58]. In the following years, a number of proteinaceous receptors were reported to have roles in AAV2 binding and transduction (e.g., fibroblast growth factor receptor 1 (FGFR1) [91], αVβ5 and α5β1 integrin [92,93,94], hepatocyte growth factor receptor (HGFR) [95], and laminin receptor (LR) [96]) but, later, could not be validated as essential factors in transduction [97,98,99]. While host factors important in the HSPG biosynthetic pathway were shown to be important for AAV2 transduction in an unbiased genetic screen [99], AAV2-like isolates from children failed to bind to HSPG, most likely due to mutations in R585 and R588 [100]. However, they retained infectivity in vivo, albeit with altered tropism [101]. This raised the possibility that HSPG binding may be a culture-acquired trait in most AAV2 isolates. In fact, Cabanes-Creus and colleagues observed that, over multiple rounds of passaging in naïve cells, natural AAV2 regained HSPG-binding affinity, which, in turn, considerably attenuated its hepatic tropism, making a strong argument that natural AAV2 lacks a strong HSPG affinity [102,103]. Besides AAV2, several other serotypes (i.e., AAV3, AAV6, and AAV13) have also been reported to use HSPG as a primary receptor [104,105]. In the case of AAV3, its HSPG binding is weaker than that of AAV2 and is mediated by an arginine residue at position 594, a site distinct from that of AAV2 [106]. Interestingly, unlike AAV2, mutating this key AAV3 residue results in the sharp loss of both cellular attachment and transduction.

AAV1 binds to α2-3 *N*-linked sialic acid (SIA) [107] through 11 amino acid residues located at the base of the three-fold spikes [119,120]. Of these, six (N447, S472, V473, N500, T502, and W503) mediate direct contact with SIA, and the other five (S268, D270, N271, Y445, and G470) play an indirect role in this interaction [119]. AAV6, which is a natural hybrid between AAV1 and AAV2 [121,122], can bind to both α2-3 and α2-6 *N*-linked SIA [107] as well as heparin [123,124]. Interestingly, AAV6 differs from AAV1 in only six amino acids, with five of these being located at the three-fold axis of symmetry and one being R585, which is involved in HSPG binding in AAV2 [52]. Also, AAV6 is 3-fold more efficient at transducing lung epithelium cells than AAV2 [123], hinting at the critical functional importance of these six residues. More importantly, after comparing the AAV1 and AAV6 crystal structures and performing functional assays, it was found that K531 confers heparin binding and gives AAV6 an advantage over AAV1 during transduction. SIA are also used as receptors by AAV4 [112] and AAV5 [125]. AAV4 was demonstrated to use α2-3 *O*-linked SIA for transduction [112]. Interestingly, knocking out Furin, a cellular endonuclease, markedly increased the localization of *O*-linked sialoglycans on the cell surface and perinuclear areas and resulted in higher viral binding, uptake, TGN localization and transduction via an unknown mechanism [126]. AAV5, the only serotype isolated directly from human clinical samples [127] and the most genetically divergent from all the other serotypes [128,129,130], requires α2-3 *N*-linked SIA for cellular binding [112]. Interestingly, when AAV5–SIA binding was eliminated by mutating leucine 587 to threonine, it lost its ability to transduce lung tissue, and its transduction efficiency in salivary glands and muscle increased [131]. While the primary receptors for AAV7 and AAV8 are still unclear, AAV9 has been described to use the terminal *N*-linked galactose of SIA for cellular binding [115,116]. Five amino acid residues (N470, D271, N272, Y446, and W503) that form a pocket at the base of the three-fold axis of symmetry are key to AAV9–galactose binding and tropism [115,116]. Finally, AAVrh.10 has been shown to bind to sulfated *N*-acetyllactosamine (LacNAc), a glycan with terminal galactose, via a pocket located on the three-fold capsid protrusions [118,132].

Like AAV2, additional co-receptors were also found for some of the other serotypes. FGFR1 [110], HGFR [111], and LR [96] were reported to be co-receptors for AAV3, the platelet-derived growth factor receptor (PDGFR) [113] for AAV5, and the epidermal growth factor receptor (EGFR) [114] for AAV6, and a putative integrin was identified for AAV9 [133]. Also, LR was reported to be a receptor or co-receptor for AAV8 and AAV9, respectively [96]. However, as was the case for many of the reported AAV2 co-receptors, direct evidence for the physical interaction between them and their respective AAV serotypes and significant differences in transduction in their absence could not be established [134]. Three independent genetic screens also yielded no evidence of their involvement in the transduction for these serotypes [99,135,136].

### 3.2. AAV Endocytosis

Clathrin-mediated endocytosis (CME) was one of the first endocytic pathways described for AAV [137,138]. Overexpression of a dominant-negative mutant of dynamin, a critical component for a number of endocytic pathways including CME, inhibited AAV2 uptake and transduction by up to 70% [137]. Paradoxically, the knock down of the clathrin heavy chain in HeLa cells [139] or the inhibition of CME with chlorpromazine [47] failed to significantly reduce transduction, suggesting that CME was not the major pathway for AAV2 uptake, at least under the conditions tested. Interestingly, the inhibition of dynamin-dependent endocytosis with dynasore failed to inhibit AAV2 uptake in HEK293T cells and, instead, showed a trend towards increased transduction [47]. In addition, the knock down or overexpression of a dominant-negative mutant of GRAF1, the most important mediator of the CLathrin-Independent Carriers and GPI-Enriched Endocytic Compartment (CLIC/GEEC) endocytic pathway [140], or the use of the CLIC/GEEC inhibitor EIPA, strongly inhibited AAV2 uptake and transduction [47]. Furthermore, critical components of this pathway (membrane cholesterol, Arf1, and Cdc42) were shown to be crucial in the formation of endocytic vesicles for AAV uptake [47]. Strikingly, the simultaneous treatment of cells with both dynasore and EIPA blocked infection and viral uptake. These data suggest that both dynamin-dependent and CLIC/GEEC uptake routes are used by AAV for endocytosis but that the CLIC/GEEC pathway results in the most productive transduction, at least in HeLa and HEK293T cells [47,141,142].

Having multiple endocytic routes can also be observed for other AAV serotypes. For example, AAV5 has been reported to have multiple entry pathways: a CME route and caveolae-mediated endocytosis [143]. AAV4, AAV5, and a bovine AAV (BAAV) can transduce epithelial cells or pass right through them on a polarized epithelial cell layer via transcytosis [144]. Interestingly, when transcytosis is blocked, transduction efficiency increases.

### 3.3. Intracellular Retrograde Trafficking

Following endocytosis, intracellular vectors face two fates: they either successfully transduce the host cell or traffic for degradation. For a successful transduction, the incoming vector traffics through the Golgi structure [136,145,146,147,148] and, within hours post entry, it accumulates in the perinuclear area [138], with different serotypes arriving at different paces [149]. It was initially thought that, from endocytic vesicles, depending on the vector dose, AAV could reach the Golgi either via the early and late endosome to the trans-Golgi network (TGN) or via the early and recycling endosome to the TGN canonical routes [150]. However, further studies disrupting key mediators of these two pathways or the SNARE protein syntaxin 5 [151] demonstrated that successful transduction involves the transport of AAV particles from the endoplasmic reticulum to the TGN [147]. Most importantly, this non-canonical trafficking route was shown to be critical for the transduction of all tested serotypes (AAV1-9) across multiple cell lines and primary cells [147].

Carette and colleagues performed a haploid genetic screen to identify host factors that are important for AAV transduction, and a 150 kDa protein named KIAA0319L was identified as the most prominent one [99]. KIAA0319L, commonly referred to as AAVR [83], localizes itself in the Golgi during the steady state and is essential for the transduction of all major AAV serotypes (except for AAV4 and AAVrh32.33) in vivo and in vitro [99,108]. Interestingly, even in AAVR knockout cells, AAV genome can still be detected, suggesting that AAVR is not involved in virion binding or uptake but plays a post-attachment role (possibly during the trafficking or escape of the virion into the cytosol) [82,108]. The structural analysis of AAVR revealed that the glycoprotein has a MANEC (motif with eight cysteines) domain, five polycystic kidney disease domains (PKD1-5), and a C-terminal transmembrane region [99]. PKD1-5 were identified as being important for AAV transduction. While mainly the AAVR PKD2 domain binds to AAV2 and AAV9 at the two-fold axis toward the three-fold axis [152,153,154], the PKD1 also shows some contribution [155]. In contrast, only PKD1 binds to AAV5, with the N-terminus interacting at the five-fold axis toward the two-fold axis, which is what the C-terminus binds to [155,156,157]. Another important factor during AAV trafficking is GPR108, which was identified as a critical host factor for transduction (but not for binding) for all AAV serotypes (except for AAV5) in two independent genome-wide CRISPR screens [135,136]. Interestingly, GPR108 functions independently of AAVR, since the AAVR-independent AAV4 and AAVrh32.33 serotypes require GPR108 [135]. Finally, another genetic screen identified TM9SF2, a Golgi protein important for transduction for all AAV serotypes [136].

### 3.4. Endosomal Processing and Escape into the Cytosol

During the transduction process, the AAV capsid undergoes conformational changes while trafficking through the endosomal system due to a drop in pH and, likely, other processes [138,147,148,158,159,160]. This conformational change involves the extrusion of the N-termini of VP1 and VP2 from the interior of the capsid [37,84,85,161]. As in autonomous parvoviruses [162], AAV2 VP1 contains a PLA2 catalytic domain [37] that plays a crucial role in enabling escape from the endosomal system into the cytosol, a necessary step for successful transduction [161]. Interestingly, for successful transduction, an extruded VP1 region is also required in the nucleus and, possibly, in the cytosol. Specifically, it has been shown that the microinjection of anti-VP1 antibodies into the nucleus of cells infected with AAV2 prevents transduction. Furthermore, the nuclear injection of a heat-treated vector, which artificially exposes VP1, results in higher levels of transduction than a vector that has not been heat-treated [85].

### 3.5. Nuclear Entry, Uncoating, and Second-Strand Synthesis

After escaping into the cytosol, intact AAV particles enter the nucleus through the NPC [91,93,163,164,165,166,167], a step described as rate-limiting [138]. In AAV2, two BR within the VP1/VP2 common region act as NLS [35,85] and have been reported to ensure its entry by interacting with Importin ß1 [86]. Surprisingly, the intact virion travels through the nucleolus, which acts as a transit station for AAV virions [163]. For example, knocking down two nucleolar proteins (nucleophosmin and nucleolin) enhanced transduction, demonstrating that they are critical factors for trafficking into and out of the nucleolus, respectively [168,169]. Although the uncoating mechanism is still under debate, a recent study showed that the AAV genome is released into the nucleolus in a stepwise process, similar to autonomous parvoviruses such as B19 [170] and the minute virus of mice (MVM) [171], which release their genome through the pores at the five-fold axis of the capsid [172]. Importantly, different AAV serotypes packaging the same genome show different release dynamics and transduction efficiencies, both in vivo and in vitro [165,173,174]. After uncoating, the AAV ssDNA is used as a template to generate a second strand, which is yet another rate-limiting step during transduction [175,176]. Next, the double-stranded AAV genome circularizes in a mostly head-to-tail fashion to form episomal monomers, and their intermolecular recombination results in high-molecular-weight concatemers, which enable long-term transgene expression [177,178,179,180,181,182,183,184,185,186,187]. Importantly, the AAV genome interacts with histones to form unique extrachromosomal chromatin structures [188,189] that are important both for in vivo episome persistence and transcriptional regulation [190]. Transcriptional regulation takes place by means of the HUSH complex and the DNA-binding protein NP220 via the epigenetic regulation of the bound histones [191] and can be influenced by the capsid and host species [191,192]. In rare cases, the AAV genome can also integrate into the host DNA at a very low frequency [87,89].

## 4. Novel AAV Variants for Targeted Tropism

While different serotypes may show different degrees of tropism toward tissue types, AAVs have a rather broad transduction profile, with no clear specificity for tissues/cell types and with overlapping tropism across serotypes (reviewed in detail by Issa and colleagues [117]). Despite their lack of specificity and off-target tissue transduction, natural AAV serotypes have been used in a number of clinical studies. AAV2 has been used to treat cystic fibrosis [193,194] and deliver neurterin, GAD, and AADC genes for Parkinson’s disease [195,196,197]. AAV4 has been used to deliver the RPE65 gene into RPE cells, providing clinical benefit [198]. AAV5 is currently been used in ongoing clinical trials to target the CNS for Huntington’s disease and the eye for X-linked retinitis pigmentosa [199], and AAV6 is being tested for hemophilia A (NCT03061201 [200]). AAV8 has been used in several trials such as the phase I/II clinical trial for X-linked retinitis pigmentosa, which showed sustained visual improvements in 6 out of 18 patients [201], and X-linked myotubular myopathy and glycogen storage disease type Ia [199]. AAV9 has been used in a number of trials to treat patients with Mucopolysaccharidosis type I, II, and IIIA, Parkinson’s disease, Gaucher disease, and neuronal ceroid lipofuscinoses (NCL)1 [199]. Finally, AAVrh.10 has been used in clinical trials for hemophilia B and mucopolysaccharidosis type IIIA [199,202].

While AAV is currently the most popular vector for in vivo gene therapy, the field is not completely free of concerns. A meta-analysis performed on data gathered from 255 clinical trials using AAV vectors reported that 18 had been put on hold, at least temporarily [203]. Most importantly, adverse side effects were observed in 78 trials, and deaths were reported in 11 trials. Four of those tragic deaths occurred in a trial to treat X-linked myotubular myopathy (XLMTM) with an AAV8 vector, and one of them occurred in a trial for Duchenne’s muscular dystrophy with an AAV9 vector [204]. Of note, two of the patients who died had been treated with ZOLGENSMA^TM^ [205]. It is important to point out, however, that in all the trials during which patients died, very high vector doses were used. Although none of these deaths have been firmly associated with the treatment, a reduction in vector dose could mitigate the safety risks, at least in some cases.

**Figure 4:**
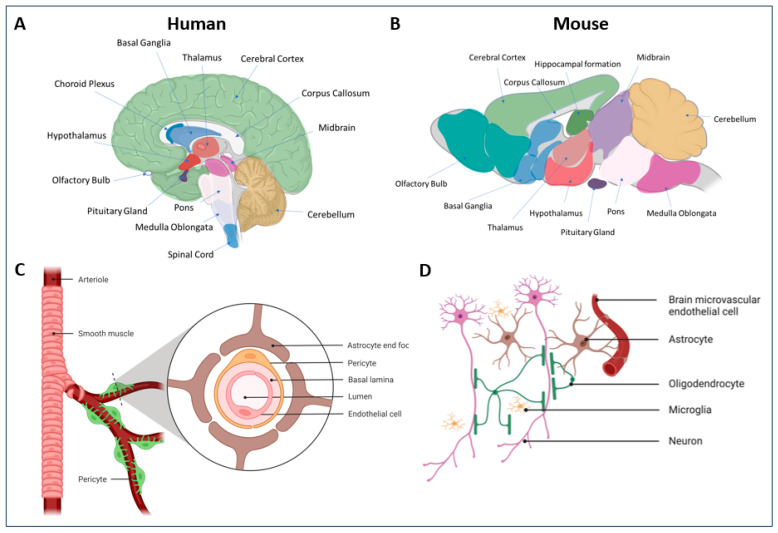
Location of anatomical structures of the human (**A**) and mouse (**B**) central nervous systems (CNSs). (**C**) Depiction of the brain vascular system with a zoomed-in cross-section of a vessel and the cell types composing it or adjacent to it. (**D**) Predominant cell types of the CNS. Images created with Biorender (available at https://www.biorender.com).

The main reason for the need of high vector doses is the relatively broad tropism of natural AAV vectors and their limited efficiency in reaching the target cells with specificity [117]. Furthermore, AAV vectors based on natural serotypes must overcome the multiple rate-limiting steps mentioned in Section 3 that can dramatically limit transduction efficiency, such as engaging with receptors which enable entry through productive trafficking pathways for successful transduction, entering into the nucleus through the NPC, undergoing second-strand DNA synthesis, and forming concatemers and nucleosomes for productive transgene expression. Further understanding of the intricate mechanisms and pathways governing these processes is pivotal in optimizing vector dosing and mitigating potential safety risks in clinical applications. This is further exacerbated by the fact that certain organs such as the CNS or the eye pose additional hurdles for the vector to penetrate and reach the target cells. For instance, to penetrate the CNS parenchyma, AAV vectors administered intravenously (IV) must cross the BBB, a complex structure composed mainly of brain endothelial cells forming tight junctions along with pericytes, astrocytic end-feet, and the basal lamina which prevents the passage of large molecules, including AAV vectors [206,207] (Figure 4C), to the CSF and brain parenchyma. On the other hand, direct injections into specific CNS areas to bypass the BBB require invasive surgical intervention and, for better or ill, enable poor coverage of large or dispersed brain regions. Intra-cerebrospinal fluid (CSF) delivery allows the bypassing of the BBB but is moderately invasive and lacks cell specificity [208,209]. Focused ultrasound is a minimally invasive technique employed with significant success to facilitate AAV vectors crossing the BBB [210]. IV administration is currently the preferred approach, but, when using natural AAV vectors, it mainly results in the transduction of peripheral organs, and vectors fail to efficiently cross the BBB and reach the CNS [211]. Furthermore, most natural AAV serotypes are sequestered by the liver, the largest internal organ, which, due to the large vector doses needed, can lead to toxicity. Finally, the need to produce large amounts of vector to accommodate the large doses required results in high production costs.

Although AAV, in general, has a favorable immune response profile, pre-existing neutralizing antibodies (NAbs) against the capsid of natural AAV serotypes are a major concern [212,213,214], since they can limit patient eligibility for AAV products. A possible way to address this issue is using AAV serotypes that are less common among humans [215] or using engineered AAV capsid variants with a lower immunogenicity and a higher specificity. In addition to the immune responses toward the capsid, the host cells also recognize AAV DNA via Toll-like receptor 9 (TLR9) to induce innate immunity and a stronger adaptive immune response [216] or via antigen cross-presentation by MHC class I or II, following intracellular proteasomal degradation of AAV capsids or therapeutic proteins to induce a cytotoxic immune response [217,218,219]. Together, these immune responses can limit the longevity of successful therapy [220]. Decreased ubiquitination and proteasomal degradation of capsids have been reported to result in a lower peptide presentation and mitigate cellular immune response [2,221,222].

The engineering of AAV capsids has become the approach of choice of many researchers to overcome the above limitations, and different engineering strategies have been employed, resulting in novel AAV capsid variants highly enriched for a wide variety of tissues upon intravascular administration (Supplementary Table S1). Among them, rational design, computationally designed ancestral AAV capsid reconstruction, in silico machine learning-guided engineering, directed evolution through error-prone PCR, *cap* gene shuffling across serotypes, or combinatorial insertion/replacement libraries are the most popular ones [2,5,223,224,225]. These approaches exploit the receptor–protein binding and intracellular trafficking properties of existing serotypes [117] and/or generate novel binding properties de novo. The preferred choice for AAV capsid engineering to manipulate virus tropism and immune recognition are the VRs due to their flexibility in accepting peptide insertions and amino acid substitutions without disrupting capsid assembly or essential trafficking mechanisms [41,226,227,228,229]. In addition, since specific residues involved in the direct binding to receptors have been identified within the VRs, engineering at those positions has allowed researchers to simultaneously re-target AAV vectors and de-target them from their natural target tissues through breaking their interactions with natural receptors. For example, this strategy has been employed to re-target AAV2, where the heparin-binding motif (R484, R487, K532, R585, and R588) has been used for peptide insertion primarily at AA587-588, whilst also disrupting HSPG engagement and, thus, facilitating tissue de-targeting [230,231,232].

### 4.1. Directed Evolution

Directed evolution is a receptor-agnostic strategy based on the generation of AAV libraries harboring mutations of the *cap* gene that confer the AAV with novel tropism for unknown receptors on target cells. The mutated AAV capsids can be generated by error-prone PCR, which introduces random point mutations [233], gene shuffling (DNA recombination between different AAV serotypes to generate a genetically chimeric capsid [234]), domain swapping (replacement of VRs between serotypes to create chimeric AAVs [235]), or using degenerate oligonucleotides to generate random peptide insertions or substitutions. This highly diverse library of mutants is screened for enhanced properties through multiple rounds of selective pressure and enrichment in vitro or in vivo.

#### 4.1.1. Peptide Insertion for CNS Targeting

**Figure 5:**
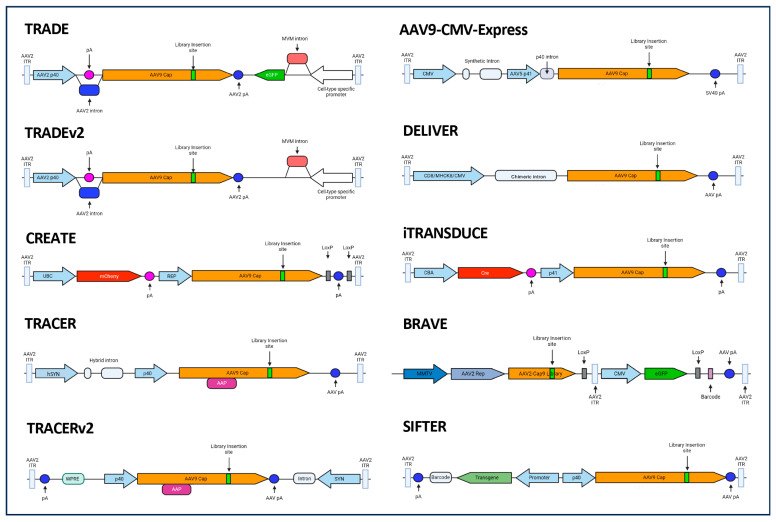
Schematic representing the genome arrangement of the library constructs of various AAV capsid engineering platforms. Images created with Biorender (available at https://www.biorender.com).

Cre recombination-based AAV targeted evolution (CREATE), a platform based on the selective recovery of capsid viral genomes from the nucleus of target cells expressing Cre recombinase in vivo or in vitro during screening campaigns [236], enabled the isolation of novel AAV variants with altered tropism [1,236,237] (Figure 5). Employing this novel technology using AAV9 as a base capsid, the AAV-PHP family of capsids emerged, with **AAV-PHP.B** exhibiting a markedly improved tropism for various tissues in mice and the capacity to access target cell populations which are challenging to reach due to their location (e.g., sympathetic, nodose, dorsal root, and cardiac ganglia) or those which are widely distributed (e.g., enteric nervous system) following IV delivery [236]. Importantly, whilst AAV9 is able to cross the BBB with a very low efficiency via trans-endothelial trafficking to the basolateral compartment [238,239], AAV-PHP.B more efficiently crossed the BBB and reached the CNS and peripheral nervous system (PNS) with broad distribution and slightly reduced liver vector genomes in adult mice (strain-dependent [240,241,242] (see Supplementary Table S1)) and rat [236,243]. AAV-PHP.B showed 40 to 60-fold greater efficiency than AAV9 at transducing the mouse CNS [236,237,240], 40 to 92-fold increase in vector genomes across CNS regions and a 75-fold increase in the spinal cord, and levels in heart and skeletal muscle similar to AAV9 [236]. Interestingly, while AAV9 preferentially transduces astrocytes when delivered IV to adult mice and non-human primates (NHP) [211,239], AAV-PHP.B transduced oligodendrocytes, neurons throughout the brain, and endothelial cells but not microglia [236,237] (for a reference to the cell types within the CNS, see Figure 4D). In the neonate rat cerebellum, AAV-PHP.B showed 2.4-fold higher transduction over AAV9 after IV delivery, and intracerebroventricular (ICV) administration into adult rats resulted in transgene expression across CNS regions and the spinal cord, demonstrating a translation of vector performance beyond mice [243] (for a reference to CNS anatomy, see Figure 4A,B).

Further engineering efforts re-diversifying AAV-PHP.B resulted in **AAV-PHP.eB**, which has the same peptide insertion as AAV-PHP.B but has additional flanking substitutions [237]. This variant efficiently transduced the mouse CNS, showing a transduction of 55–76% of neurons depending on the region, a >2.5-fold increased transduction compared to AAV-PHP.B, and similar percentages of glia. High CNS tropism was also observed in rat [237,242]. To de-target AAV-PHP.eB from the liver, further screening campaigns were performed, where additional VRs such as the VR-IV were modified [244]. In addition, the CREATE technology was refined to normalize variant NGS read scores from the tissue to input administered variant distribution (enrichment), generate a synthetic oligo pool of library variants selected at each screening round to reduce propagation bias, and include codon replicates of each variant within the pool to reduce the rate of false positives, which was called Multiplexed-CREATE (M-CREATE) [244]. Two rounds of screening of M-CREATE in mice generated **AAV.CAP-B10** (AAV-PHP.eB with VR-IV substitutions), which retained mouse CNS targeting but exhibited a decreased transduction of peripheral organs and a 50-fold decreased liver tropism following IV delivery compared to AAV-PHP.eB or >100-fold compared to AAV9 [245]. AAV.CAP-B10 demonstrated specific neuronal targeting within the CNS, with fewer transduced astrocytes and oligodendrocytes compared to AAV-PHP.eB. Importantly, AAV.CAP-B10 showed broad and robust transgene expression of the CNS in adult marmoset across regions (4-fold increase over AAV9 and AAV-PHP.eB) as well as in the spinal cord and the dorsal root ganglia (DRG), with a 17-fold-lower liver expression compared to AAV9 or AAV-PHP.eB. However, when administered as a pool in infant rhesus macaques, AAV.CAP-B10 showed a comparable, although slightly higher, enrichment in the CNS to that of AAV9 [246]. The emergence of techniques, such as single-cell RNA sequencing (scRNA-seq), capable of profiling barcoded AAV variants in a single animal across numerous complex cell types by taking advantage of transcriptomic resolution has led to a more in depth understanding of the cell type specificity of AAV variants [247]. The employment of this technology in mice after the IV delivery of capsid variants confirmed the preferential transduction of neurons over astrocytes and oligodendrocytes for both AAV-PHP.eB and AAV.CAP-B10, with differences between the preferred GABAergic and glutamatergic neuron subtypes. Another variant from the same campaign with CNS tropism in mouse, **AAV.CAP-B22**, exhibited a higher CNS transduction compared to AAV9 (12-fold) in marmoset, with more transduced astrocytes than AAV9 or AAV.CAP-B10, albeit with relatively similar levels to AAV9 in the liver [245], but failed to translate into newborn rhesus macaques, where it exhibited only a slightly higher trend in CNS enrichment compared to AAV9 when administered in a pool format [246].

**AAV-PHP.A** exhibits 2.6-to-8-fold-higher mouse CNS transduction than AAV9 after IV delivery, with a pronounced bias toward astrocytes, and a 152-fold-reduced transduction of the liver and other peripheral organs compared to AAV9 [236]. These properties result in AAV-PHP.A having a high tropism for the CNS compared to the liver, showing a CNS:liver ratio 400 to 1200-fold higher than that observed for AAV9. Importantly, like AAV-PHP.B, AAV-PHP.A is able to transduce cortical neurons and astrocytes derived from human induced pluripotent stem cells in a spheroid 3D configuration, albeit at a lower efficiency than AAV-PHP.B, showing their potential for translation into humans. **AAV-PHP.V1** is also able to cross the BBB in mice and results in 14% CNS transduction, with 60% of cortical transduced astrocytes (lower than AAV-PHP.eB), a similar transduction of oligodendrocytes compared to AAV-PHP.eB, and biased tropism toward brain vascular cells [244,247,248]. Interestingly, this variant also showed the increased transduction of human brain microvascular endothelial cells in cultures [244]. **AAV-PHP.N** achieves 26% mouse CNS transduction after IV delivery and exhibits bias toward neurons across CNS regions [244,248]. Having a toolbox of variants with the targeting of diverse cell types in the CNS is very useful for neuroscience research and disease correction studies.

With the goal of visualizing and quantifying individual AAV genomes from tissues, Wang and colleagues developed a novel application called signal amplification by exchange reaction fluorescence in situ hybridization (SABER-FISH) [249]. Applying this technique, the group found that AAV genomes would enter retinal microglia but be degraded prior to nuclear localization. More recently, a high-resolution method for the spatial transcriptomic profiling of endogenous and viral RNA in intact tissues, named Sequential Fluorescence In situ Hybridization (USeqFISH), was developed [248] and shown to be applicable to tissues when combined with the tissue-clearing technique PACT [250] (validated in mouse and NHP) or to cells in vitro. This technique enabled the simultaneous in-depth characterization of previously engineered variants, facilitating their comparison in tissue targeting, cell specificity, and expression intensity within transduced cells [248]. the Employment of USeqFISH revealed that AAV.CAP-B10 transduces the CNS with a similar efficiency to AAV-PHP.eB (48%), albeit with a preference for different CNS regions and by targeting different cell types depending on the region and tissue depth (e.g., a higher bias of AAV-PHP.eB toward inhibitory neurons and astrocytes than AAV.CAP-B10 in the cortex). AAV-PHP.V1, AAV-PHP.B8, and AAV-PHP.N also showed preference for certain CNS regions, with AAV-PHP.V1 exhibiting relative tropism toward vascular cells, and, for AAV-PHP.N, a bias toward excitatory neurons in the cortex. Interestingly, all variants showed preference for inhibitory neurons in the striatum. Moreover, a novel rationally designed variant, **AAV-PHP.AX** (AAV-PHP.eB mutant with an additional microglia-targeting peptide [251]), showed 41% CNS transduction in mice following IV delivery, with a similar CNS region enrichment bias to AAV-PHP.eB but a lower overall astrocyte transduction [248]. Interestingly, when carrying a cargo with the astrocyte-specific glial fibrillary acidic protein (GFAP) promoter, AAV-PHP.AX showed a more efficient transduction of astrocytes than AAV-PHP.eB, demonstrating that specific gene expression can also be modulated by altering the cargo.

In contrast to the AAV-PHP.B family, other AAV9 variants that were also recovered from CNS tissue and had motif patterns distant from it (**AAV-PHP.C1** and **AAV-PHP.C2**) showed BBB crossing across mouse strains after IV delivery [244]. AAV-PHP.C1 showed a similar astrocyte tropism and a lower neuronal targeting compared to AAV-PHP.B. AAV-PHP.C2 exhibited bias toward vascular cells and astrocytes and away from neurons, which indicates that this variant could be a good choice for non-neuronal targeting [247]. **AAV9-X1** showed the transduction of 65–70% of endothelial cells in the CNS across regions, which was higher than that for other endothelial variants, such as AAV-PHP.V1 and AAV-BR1 (variant further described below), which showed 40% transduction in this study [252]. Importantly, AAV9-X1 showed more specificity, with 95% of transduced cells being endothelial cells, compared to AAV-PHP.V1 (40% with the additional targeting of neurons and astrocytes) or AAV-BR1 (60% with the additional targeting of neurons). It is worth noting that endothelial cells from peripheral organs were not transduced by AAV9-X1. Interestingly, while AAV9-X1 efficiently transduced brain endothelial cells, liver transduction was minimal in the liver of BALB/c and CBA/J strains. To further de-target AAV9-X1 from the liver, the residue substitution from AAV-CAP.B10 was transferred to AAV9-X1, generating a novel variant called **AAV9-X1.1**, which further improved brain endothelial cell transduction across regions in mice to 82–85% and in rats. Additionally, the mutation of N272 or W503 to alanine to disrupt galactose binding resulted in two CNS endothelial variants (**AAV9-X1.4** and **AAV9-X1.5**, respectively) with reduced liver transduction. The transfer of the AAV9-X1 peptide into AAV1 (**AAV1-X1**) and AAV-DJ (variant further described below) (**AAV-DJ-X1**) resulted in improved mouse CNS transduction with endothelial cell targeting [252], indicating that the AAV9-X1 phenotype can be transferred to other serotypes, in contrast with what had previously been reported for AAV-PHP.B [253,254]. Importantly, in contrast to AAV-BR1, AAV9-X1, AAV9-X1.1, AAV1-X1, and AAV-DJ-X1 exhibited an improved transduction of human brain microvascular endothelial cells (HBMECs) over AAV9 [252]. Furthermore, AAV9-X1.1 showed an increased transduction on ex vivo brain slice cultures from southern pig-tailed macaque (interestingly, mainly neuronal) or human compared to AAV9, highlighting its translational potential. Interestingly, despite this, the variant showed no enhanced transduction of the marmoset CNS in vivo after IV delivery, but it demonstrated enhanced transduction of the neonate rhesus macaque CNS across regions (98% of the transduced cells being neurons, with a small proportion of endothelial or glial cells) and reduced liver, muscle, and enteric system transduction compared to AAV9. **AAV-AS** is a variant based on **AAV9.47** (comparable CNS tropism to AAV9 but decreased liver transduction [255]) in which 19 alanine residues were inserted after the first residue of VP2 [256]. This novel variant showed a higher CNS transduction than AAV9 after IV delivery into mice and comparable vector genome levels in the liver, muscle, lungs, pancreas, and kidneys. Also, it reached 36% of neurons, glia, and endothelial cells in the CNS and spinal cord, unlike AAV9, which only transduced glia and endothelial cells in the CNS. Interestingly, AAV-AS exhibited a comparable transduction profile in cats, with broad neuronal targeting in the CNS and spinal cord, and transduced only a minimal number of glial cells and no endothelial cells. Another study generated **AAV r3.45**, an AAV2 variant which retained HSPG binding and was able to transduce rat, murine, and human neural stem cells (NSC) in vitro with more selectivity than parental AAV2 or AAV6 [257,258]. After intracranial injection in rat or mice, it transduced preferentially NSCs with a higher efficiency than the parental serotype [258]. Finally, a recent study engineered **AAV-PHP.eC**, a novel variant derived from AAV-PHP.C1 which outperformed AAV-PHP.C2 in CNS targeting in multiple mouse strains after IV delivery, transducing neurons and astrocytes [259].

More recently, the employment of the AAV9-CMV-Express platform, which relies on the recovery of library transcripts to ensure the selection of transcriptionally functional variants [260] (Figure 5), led to the identification of the AAV9-derived variant **AAV-BI30** [261]. This variant showed an overall higher, broader, more selective, and strain-compatible transduction of endothelial cells in vitro and in vivo compared to previously identified variants such as AAV-BR1. AAV-BI30 targeted 84% endothelial cells within the mouse CNS after IV administration, reaching arteries, veins, and capillaries, as well as endothelial cells from peripheral organs such as the eye (69–81%), spinal cord (76%), lung, heart, and kidney [261]. AAV-BI30 showed more specificity for brain endothelial cells in the CNS than AAV-PHP.V1, although it also exhibited a robust transduction of the liver. Importantly, AAV-BI30 showed a similar transduction profile for brain endothelial cells in mice and rats and transduced immortalized human cerebral microvascular endothelial cells (hCMEC/D3) and human and mouse brain microvascular endothelial cells (BMVEC) in vitro more efficiently than AAV9, indicating potential for translatability.

Most recently, engineering efforts on AAV9 in adult marmoset led to the generation of **AAV.CAP-Mac** and **AAV.CAP-C2** [246]. To assess translatability, these two variants were administered into infant and adult rhesus macaques, infant African green monkeys, and adult marmoset via IV or ICM delivery. When delivered as a pool into infant rhesus macaque via IV delivery, both variants showed higher RNA enrichment in the CNS compared to AAV9 (6-fold for AAV.CAP-Mac and 4-fold for AAV.CAP-C2) and lower liver tropism. When administered individually, AAV.CAP-Mac transduced multiple regions of the CNS and primarily targeted neurons and astrocytes. In contrast, ICM delivery resulted in a decreased transduction of the CNS and a low reach to subcortical structures. In infant African green monkeys, AAV.CAP-Mac showed a broad and stronger transduction of the CNS than AAV9 after IV delivery, similar to that observed in rhesus macaques, with bias toward neurons, except in the thalamus, where it transduced marginally higher numbers of astrocytes. In peripheral organs, AAV.CAP-Mac was comparable to AAV9. Interestingly, when assessing BBB penetrance in adult individuals, AAV.CAP-Mac’s expression in rhesus macaques was evident only in certain CNS regions, in contrast to that observed in infant rhesus macaque, and, for the first time, CNS expression in marmoset appeared to be primarily biased towards the vasculature. In adult mice, AAV.CAP-Mac showed neuronal bias when delivered ICV but primarily transduced the vasculature when delivered IV, suggesting that AAV.CAP-Mac is unable to cross the adult mouse BBB to access the CNS [262]. However, transduction was observed in neurons, astrocytes, and the vasculature upon IV administration into neonatal mice. These studies show how AAV.CAP-Mac transduction varies in spread across CNS regions and cell specificity across species, ages, and delivery routes. Finally, this variant exhibited a 45-fold increase in transduction efficiency over AAV9 and a higher intensity of human neurons derived from pluripotent stem cells in cultures [246].

Other screening platforms such as iTransduce have also led to the selection of novel variants with targeted tropism (Figure 5). iTransduce is based on the use of Cre recombinase within the viral vector library to activate the expression of a reporter gene contained in transgenic mice (Ai9 mice carrying a floxed stop tdTomato cassette) [263]. Flow cytometry and the cell sorting of transgene-expressing cells enable the recovery of only cells containing transcriptionally functional variants whose genomes can then be isolated. The employment of this technology in AAV9 libraries led to **AAV-F**, a novel variant which after IV delivery into mice transduced the CNS across regions with a 119-fold increase over AAV9 as well as the spinal cord, albeit with comparable genome levels in the liver [263]. This variant transduced 40.78% of astrocytes (65-fold higher than AAV9 and 1.44-fold higher than AAV-PHP.B) and 6.67% of neurons (171-fold higher than AAV9 and 1.58-fold lower than AAV-PHP.B), including excitatory and inhibitory neurons and motor neurons in the spinal cord. Importantly, unlike AAV-PHP.B, AAV-F transduced the CNS across mouse strains, showed CNS and spinal cord transduction also via intrathecal injection into the spinal cord and intraparenchymal injection, and mediated a 3-fold-higher transduction of primary human stem cell-derived neurons in a culture compared to AAV9, highlighting its translational potential. When tested in cynomolgus macaque following lumbar intrathecal injection, AAV-F exhibited broad CNS transduction, an overall higher transduction than AAV9 for motor neurons and interneurons in the spinal cord, with a comparable, low astrocyte transduction, the targeting of Schwann cells in the sciatic nerve, a lower neuronal transduction in the DRGs than AAV9, and lower levels of vector genomes than AAV9 in the liver (4.87-fold), spleen (3.85-fold), and heart (2.17-fold) [264].

In order to reduce the number of non-functional and off-target AAV capsid variants recovered during screening campaigns, the powerful technology TRACER (tropism redirection of AAV by cell-type-specific expression of RNA) enabled the recovery of a bulk capsid library of RNA expressed in a cell type-specific manner from non-transgenic animal tissue [265] (Figure 5). By employing this technology, variants with similar AA motifs to AAV-PHP.C2, AAV-F, or AAV-PHP.eB were identified, as well as alternative motifs at AA586 [265]. Interestingly, different peptide motifs showed bias for different cell types, such as astrocytes or neurons, were strictly restricted to the C57BL/6 mice strain, or enabled the transduction of mouse BMVECs in cultures, suggesting that different families of variants may bind to different receptors or use distinct mechanisms to cross the BBB in mice. These screening efforts led to the characterization of 10 AAV9-based capsid candidates (**9P03**, **9P08**, **9P09**, **9P013**, **9P016**, **9P031**, **9P032**, **9P033**, **9P036**, and **9P039**). All novel variants exhibited broad transduction of the mouse CNS, with some being predominantly biased toward neurons (9P31, 9P32, 9P33, 9P36, and 9P39) and others toward non-neuronal cells (9P03, 9P08, 9P09, 9P13, and 9P16), demonstrating their potential for diverse disease applications. Several variants showed similar CNS targeting to AAV-PHP.eB (9P08, 9P16, 9P33, and 9P36). However, 9P31 outperformed AAV-PHP.eB, with transduction up to 385-fold over that of AAV9 in the CNS, 1000-fold in the spinal cord and 5-fold in the heart. Variants 9P09, 9P33, and 9P39 showed 100-fold de-targeting from the liver.

A recent study generated AAV9derived **AAV-Se1** and **AAV-Se2**, which exhibited enhanced broad CNS targeting and 3-fold or 8-fold lower liver transduction, respectively, across mouse strains following IV delivery [266]. Interestingly, while AAV-Se1 transduced mainly endothelial cells and some sparse astrocytes, AAV-Se2 exhibited a neuronal and astrocytic bias, with a 2-fold-lower transduction of neurons than AAV-PHP.eB and a slightly higher transduction of astrocytes. In marmosets, these variants maintained CNS targeting across regions (2.2-to-4.7-fold over that of AAV9), with specificity for neurons and astrocytes, and showed reduced liver, muscle, and heart transduction following IV delivery. Importantly, both variants transduced human neuronal cultures in vitro.

Capsid engineering of AAV2 has also generated capsid variants able to cross the BBB after intravascular delivery in mice and transduce the CNS. A good example is **AAV-BR1**, which mediated a 650-fold-higher transgene expression in the CNS than AAV2 across regions (primarily brain endothelial cells and some sparse neurons) and spinal cord and the undetectable transduction of the liver or heart following IV delivery into mice [267]. Importantly, the transduction of primary cerebral microvascular endothelial cells (PCMECs) and hCMEC/D3 cells was also confirmed.

Directed evolution has also been applied to screen AAV variants able of retrograde transport from the axonal terminals of neurons toward the cell nuclei. AAV2-derived **rAAV2-retro** demonstrated retrograde transport to several regions of the mouse CNS after intraparenchymal injection, with a 133-fold-higher transduction than AAV2 and efficiencies even higher than those of synthetic retrograde tracers [268]. This novel variant has a great potential in neuroscience research to interrogate neuronal circuit function and enables the administration of vectors in small strategic locations to reach larger areas of the CNS. Additionally, rAAV2-retro was administered to the musculature of neonatal mice and showed a 57% specific transduction of lower motor neurons projecting to the muscle, and, through retrograde transport, rAAV-retro achieved transduction of the spinal cord and brainstem, with some differences between sub-regions in both, and of the DRGs [269]. Interestingly, the authors demonstrated rAAV-retro’s ability to diffuse through the spinal nerve into the CSF and, also, from the blood vessels after intramuscular injection into the CSF, ultimately enabling it to transduce neurons in the spinal cord and reach the CNS.

#### 4.1.2. Peptide Insertion for PNS Targeting

Screening campaigns for AAV9 have also been carried out to target the PNS and have resulted in variants such as **AAV-PHP.S**, which displays improved tropism for neurons within the PNS and organs including the gut and heart in mice after IV administration [237]. This variant transduced 82% of DRG neurons, cardiac and enteric neurons of the small intestine and colon, and astrocytes of the enteric nervous system, to a smaller extent, and exhibited the robust transduction of the liver, lungs, heart, and stomach, at a high dose. **AAV-MaCPNS1** and **AAV-MaCPNS2** were also evolved from AAV9 to transduce the PNS of mouse, rat, and NHP [270]. In mice, they transduced neurons of the nodose ganglia (28–35%) and the DRG (18–16%) of the spinal cord with a ~2-fold-higher efficiency than AAV9 or AAV-PHP.S following IV delivery. Interestingly, AAV-MaCPNS2 transduced the enteric ganglia in the small intestine 2-fold higher than AAV9 as well as the large intestine at higher viral doses. On the other side, AAV-MaCPNS1 exhibited a 1.5-fold-lower liver transduction compared to AAV9 or AAV-PHP.S, suggesting a potentially safer profile. In rats, the systemic delivery of AAV-MaCPNS1 and AAV-MaCPNS2 recapitulated the PNS tropism observed in mice, showing the efficient transduction of sensory, sympathetic, and parasympathetic ganglia and enteric neurons, with slight changes in neuronal specificity, and showed no expression in the liver. Similar to that observed in rodents, AAV-MaCPNS1 and AAV-MaCPNS2 exhibited enhanced transduction in the PNS (DRG, small intestine, and the spinal cord) in adult marmoset after IV delivery but also in the CNS across regions (neurons and astrocytes). AAV-MaCPNS2 displayed a 5.5-fold increase in neuronal transduction and a 25-fold increase in astrocytic transduction over AAV9 in the cortex, and AAV-MaCPNS1 displayed a 4-fold increase in transduction for cortical neurons. In infant rhesus macaque, both variants also showed enhanced transduction in the PNS (spinal cord, DRG, and gastrointestinal tract), with mainly neuronal targeting, and in the CNS, with a broad transduction of neurons (AAV-MaCPNS1 and AAV-MaCPNS2) and astrocytes (AAV-MaCPNS2) but not oligodendrocytes or endothelial cells. Together, these data show AAV-MaCPNS1 and AAV-MaCPNS2 as vectors with translational potential for targeting the PNS, with slightly different cell specificities and, potentially, safety profiles.

#### 4.1.3. Peptide Insertion for Eye or Ear Targeting

The eye has also been a target for AAV gene therapy, generating variants such as the AAV2-derived **7m8**, which has a peptide insertion in VR-VIII which disrupts basic arginine residues in the VR-IV implicated in HSPG binding [231]. When injected intravitreally into adult mice, 7m8 resulted in the broad transduction of the retina, reaching various cell types and deep layers including the retinal pigment epithelium (RPE), in contrast to AAV2, which only reached cells in the surface layers. When administered to cynomolgus macaque, 7m8 showed a significantly higher expression in the retina and inside the fovea compared to the control vector, reaching deep layers but not the RPE. This could be explained by the thicker inner-limiting membrane (ILM) in NHP compared to rodents, which poses a harder physical barrier to reach target cells. A novel scRNA-Seq-based approach named scAAVengr, involving the simultaneous sequencing of cellular and viral transcripts in vivo across different cell types in tissue, enabled researchers to quantitatively assess the efficiency and specificity of newly engineered AAV vectors [271]. Employing this approach in canines, the novel AAV2 variant **K912** was recovered from the deeper layers after intravitreal injection. When intravitreally injected into marmosets and cynomolgus macaque in a pool format, it demonstrated a high transduction of different retinal types across regions [271]. When administered individually into cynomolgus macaque via intravitreal injection, K912 showed a transduction of 2% of the total retinal cells, reaching deep layers but not the RPE.

**AAV-S**, an AAV9 variant identified from an iTransduce library [263], transduced the outer ear, nearly all cell types of the cochlea, with a robust transduction in hair cells (HC) and supporting cells (SC), and the vestibular organs when injected directly into the ears of neonatal and adult mice through the round window membrane (RWM) [272]. In cynomolgus macaque, AAV-S also effectively transduced almost all cell types of the cochlea, with some differences in terms of the targeted cell types compared to mice, and the vestibular sensory organs.

These studies demonstrate the broad reach of both AAV2- and AAV9-derived variants in targeting the various cell types forming the eye and ear and their potential for translation.

#### 4.1.4. Peptide Insertion for Heart and Muscle Targeting

In a recent study, Gonzalez and colleagues sequentially screened infant pigs of two different strains (IV delivery or into the lumbar cistern), mice (IV delivery), and adult cynomolgus macaques (IV or ICM infusion) to increase the potential translatability to humans and isolated a cross-species-compatible AAV (ccAAV) variant named **AAV.cc47** [273]. When injecting AAV.cc47 IV into mice, transduction was increased over that of AAV9 in the heart (21-fold), skeletal muscle (16-fold), liver (2-fold), and CNS (3-to-4-fold). Interestingly, ICV administration into neonatal mice resulted in a higher transduction and spread across CNS regions. In agreement, when administered ICM to adult cynomolgus macaques, AAV.cc47 exhibited robust expression in the CNS and was able to penetrate the parenchyma, showing higher neuronal and glial targeting than AAV9, as well as a higher transduction in the heart, spinal cord, and liver. In pigs, intrathecal infusion into the lumbar cistern resulted in increased transduction and spread in the CNS (neurons and glia).

The targeting of the muscle presents challenges due to its large percentage of body mass within the body, which requires the broad distribution of vectors and, thus, high doses which can lead to toxicity [274,275]. The development of muscle tropic variants has been a matter of high interest, and there are currently several AAV variants available for mice and NHP that also demonstrate the transduction of human primary myotubules. A novel variant named **AAVMYO** was identified by employing a novel bioinformatic pipeline for AAV screening using barcodes for the qualitative and quantitative tracking of the DNA and RNA levels of pooled variants, normalization strategies inter/intra-tissue for capsid performance, and the inclusion of AAV controls as benchmarks for various tissues [276]. Following IV delivery, this variant appeared to be highly enriched in the entire musculature of mice compared to AAV9, comprising skeletal muscle (17-to-50.1-fold), diaphragm (11.6-to-61-fold), and heart (5.8-to-11-fold), and was de-targeted from the liver even at high doses [276,277]. Given its translatability across mouse strains, the authors suggested that AAVMYO may translate into NHP. The addition of liver de-targeting mutations P504A and G505A into AAVMYO achieved a reduced liver transduction, albeit at the expense of the reduced transduction of the musculature.

The screening platform DELIVER (Directed Evolution of AAV capsids Leveraging In vivo Expression of transgene RNA) (Figure 5), which enables the recovery of capsid library transcripts expressed from a ubiquitous or cell-specific promoter, yielded **MyoAAV 1A** through screening in mice [278]. MyoAAV 1A demonstrated a broad and higher transduction of skeletal muscle (10 to 29-fold) and heart (6.3-fold) than AAV9, a lower liver transduction (2.8-fold), and a comparable or slightly lower transduction of lung, kidney, spleen, and CNS after IV administration into mice. When administered intramuscularly, it exhibited a 14-fold-higher expression in skeletal muscle than AAV9. Importantly, this variant transduced skeletal muscle across mouse strains as well as mouse primary myotubules and human primary myotubules with a higher efficiency than AAV9. Further engineering efforts based on structural predictions for the MyoAAV 1A VR-VIII produced **MyoAAV 2A**, which demonstrated improved properties, maintaining a low liver tropism and showing a 10-to-80-fold-higher skeletal muscle transduction and a 17-fold-higher transduction in the heart than AAV9. Also, MyoAAV 2A transduced human primary myotubules more efficiently than AAV9 and MyoAAV 1A. Furthermore, additional engineering and screening in cynomolgus macaques identified **MyoAAV 4A**, **4E**, **3A**, and **4C**, which displayed high transduction profiles for skeletal muscles, exemplifying the potential of the platform for translatability across species.

#### 4.1.5. Peptide Insertion for Lung or Liver Targeting

The vascular endothelium of the lung presents an attractive target for AAV gene therapy. AAV2-derived **AAV-VNT**, isolated via in vivo phage display biopanning following IV administration into rats, showed a 2.5-fold or an 11-fold increase in vector genomes over AAV2 in the CNS or lung, respectively [279]. Liver transduction was comparable to the control for both variants, and no expression was found in the heart or kidneys. The rating of variant performance based on tissue specificity led to the identification of an enriched sequence motif for lung-enriched variants following IV administration into mice, and **AAV2-ESGHYGF** was identified, a variant which exhibited strong and specific lung transduction (200-fold higher than AAV2) and 250-times more vector genomes in the lung than in other organs [280]. Furthermore, it selectively targeted pulmonary endothelial cells over endothelial cells in other tissues. The intra-peritoneal administration of AAV2-ESGHYGF also resulted in specific lung targeting.

Recently, a novel pipeline leveraging machine learning, called Fit4Function, has emerged as a significant conceptual and technological advance, proving successful in generating novel AAV capsid variants de novo [260]. Fit4Function is an in silico prediction model that incorporates multi-function properties to find variants optimized for several properties simultaneously. Fit4Function prediction models of in vivo performance were trained using AAV productivity data, functional assays in relevant mouse and human cell lines, and biodistribution data from mice. When narrowing down the predictive models to cross-species hepatocyte gene delivery, Fit4Function selected variants **BI151, BI152, BI153, BI154, BI155, BI156, and BI157**, which met at the intersection of both the production and functional fitness models. All the variants were produced at similar or superior yields to AAV9, outperformed AAV9 in transducing human hepatocytes in vitro, and showed a similar or superior transduction of mouse liver. When tested as a pool in adult cynomolgus macaques, the variants showed a higher enrichment than AAV9 in the liver.

#### 4.1.6. Gene Shuffling

Gene shuffling relies on the high homology of the *cap* gene between serotypes, which enables homologous recombination and, thus, the generation of novel capsid variants composed of different regions and motifs with unique targeting properties. Capsid gene shuffling generated **cA2**, a chimera of AAV1, 2, 6, 8, and 9 which showed a higher transduction of the heart, liver, and muscle than AAV2 after IV administration into mice [234]. The in vitro recombination of related parental *cap* genes from serotypes with >50% homology resulted in a higher efficiency of gene shuffling, leading to **AAV-DJ**, a chimeric variant composed primarily of the AAV2, 8, and 9 capsid sequences [232]. This variant was able to bind to HSPG and transduced various human cell systems, including primary hepatocytes, with a higher efficiency than parental serotypes. It also demonstrated mouse liver transduction equivalent to that of AAV8 and AAV9 but superior to that of AAV2 when administered IV. Heart, kidney, and spleen transduction was at similar levels to that of AAV8 and AAV9 and lower in the lungs, CNS, pancreas, and gut. AAV-DJ was also mutated (R585Q) to prevent its binding to HSPG and used as a platform for a peptide display for further engineering and screening in mice, resulting in the identification of **AAV-DJ-NSS** and **AAV-DJ-MVN**. When administered via nasal aspiration, AAV-DJ-NSS showed retargeting from endothelial to alveolar cells, while AAV-DJ-MVN targeted alveolar macrophages. In addition, a later report mutated AAV-DJ (K137R, T251A, or S503A) with the goal of reducing the proteasomal degradation of the virus during cell transduction and reported a 20–30% higher gene expression for K137R and S503A in vitro [281]. After IV delivery into mice, all three mutations resulted in a higher genome residence in the liver and a lower one in the spleen, with the S503A mutant also showing higher residence in skeletal muscle and the heart and K137R in the kidneys. No significant differences were observed in the CNS, lungs, or pancreas. This study demonstrated the relevance of manipulating virus trafficking through capsid engineering and how that can dramatically increase transduction efficiency.

AAV2.5T and AAVM41 are other success stories of *cap* gene shuffling. **AAV2.5T** is a hybrid of AAV2 and 5 with an A581T mutation, identified by screening an organotypic human airway model, and is able to specifically transduce human airway epithelia from their apical surface with more efficiency than AAV2, 5, and 9 [282]. **AAVM41** is a hybrid of AAV1, 6, 7, and 8 that exhibits a similar heart tropism to that of AAV9 but an 81.1-fold-lower liver transduction in mice after IV delivery [283]. Also, despite showing a higher skeletal muscle transduction than AAV6, its levels are significantly lower than those of AAV9, both after IV and intramuscular administration. In agreement, AAVM41 and AAV9 transduced nearly 100% of cardiomyocytes after IV delivery into hamsters, but AAVM41 showed a much lower transduction of skeletal muscle than AAV9.

More recently, SCHEMA, a structure-guided recombination technology which predicts the optimal crossover points for the DNA shuffling of chimeric proteins and, thus, enables the generation of highly diversified chimeric libraries with minimal structural disruption, was employed to generate novel capsids for targeting the CNS [284]. This technology resulted in the generation of **SCH9**, a chimera of AAV2, 6, 8, and 9 able to bind to both HSPG and galactose. This variant transduced 60% of the adult NSCs in the subventricular zone of mice after ICV and exhibited a 24-fold-higher transduction than AAV9, with higher neuronal targeting, which demonstrated its ability for retrograde transport [284]. This technology also generated **AAV-B1**, a chimera of AAV8 and rh10 and with additional single-residue changes [285]. This variant transduced the mouse CNS across regions (neurons, endothelial cells, and oligodendrocytes) and the spinal cord (endothelial cells and astrocytes) upon IV delivery more efficiently than AAV9, and reached the retinal endothelium. AAV-B1 also showed a 3.6-fold-lower genome content in the liver compared to AAV9 and a higher content in skeletal muscle, heart, pancreas, and lungs. When delivered IV to juvenile cats, it transduced neurons of the CNS across regions but no endothelial cells, alongside skeletal and cardiac muscles, and showed a negligible transduction of the liver. These studies demonstrate the power of gene shuffling in generating variants that target various cell types in the CNS, as observed with peptide insertion/substitution strategies.

**AAV-LK03** is a chimera of AAV3B, AAV1, 2, 4, 6, 8, and 9 engineered by DNA shuffling and screened in mice partially repopulated with primary human hepatocytes [286]. Interestingly, this variant showed efficient transgene expression in human but not mouse hepatoma cell lines, despite the detection of a similar number of vector genomes [192,286]. In addition, it transduced the hepatocytes of humanized mice (10-fold higher than AAV8) but not those of non-humanized controls, and it exhibited only the transduction of human hepatic tumors but not mouse hepatocytes in a hepatocellular carcinoma xenograft model [286]. In another study, Pekrun and colleagues engineered chimeric DNA-shuffled variants from human, monkey, porcine, bovine, murine, avian, and goat natural serotypes and variants such as AAV-DJ and AAV-LK03, screening them in intact and dissociated human islet cells [287]. From the screening campaign, the above authors identified **AAV-KP1** (chimera of seven serotypes and AAV3B), which penetrated and transduced primary human islet cells and human embryonic stem cell-derived β-cells with a high efficiency. In addition, it robustly transduced both mouse and human hepatocytes in a humanized chimeric mouse model following IV delivery, at higher levels than AAV-DJ.

#### 4.1.7. Error-Prone PCR

Error-prone PCR has also led to the generation of novel AAV variants with enhanced tropism. Focusing mutations on the GH-loop spanning AA390-627 of AAV9 and screening mice following intra-peritoneal injection, AAV9.24, AAV9.45, and AAV9.61 were identified as being liver de-targeted [255]. When injected IV, the variants retained similar expression levels in the heart and skeletal muscle to those of AAV9 but exhibited a 10-to-25-fold-lower liver transduction, with AAV9.45 displaying the highest tropism for cardiac tissue. In contrast, another variant called AAV9.68 showed a slightly enhanced transduction of the liver. AAV4.18 is an AAV4 variant generated via error-prone PCR that shows ~25% lower cell binding via O-linked 2,3-SIA, 10-fold-lower virus uptake into African green monkey kidney cells in vitro, rapid elimination from the bloodstream as early as 6 h post IV administration into mice, 100-fold-lower heart transduction, and 10-fold lower transduction in the lungs [288]. Despite the apparent defective phenotype, AAV4.18 was able to spread throughout the CNS parenchyma to higher levels and a broader reach than AAV4. Also, in contrast to AAV4, it selectively transduced migrating progenitors and neuroblast in the rostral and caudal directions via ICV injection in neonatal mice and exhibited transduction of the brain microvasculature (not endothelial cells or mature neurons) [289].

### 4.2. Rational Design

Rational design is based on changing the AAV capsid’s ability to bind to receptors using information on the target receptor and/or how capsid proteins could interact with it. Rational design can be achieved by mutating capsid protein amino acids, swapping the VRs between serotypes with different known binding properties, or inserting known receptor-binding peptides into the capsid. This strategy has been employed to generate novel tropism, increase the transduction efficiencies of natural AAV serotypes, and reduce immune reactivity against parental serotype/s. Some examples include **AAV2.5**, an AAV2 capsid with residues mutated to those from AAV1, able to bind to the AAV2 receptor but with AAV1 muscle tropism [290]. In rats, substantial transduction was found in the CNS upon delivery through the CSF [208]. When injected into the CSF (via cisterna magna or the lumbar cistern) of cynomolgus macaques, it exhibited a transduction profile that was similar to that of AAV9 and a 100-fold-lower number of vector genomes in the spleen. Interestingly and in contrast to its delivery via the cisterna magna, the administration via the lumbar cistern resulted in the high transduction of the DRG.

Other examples are **AAV2G9** and **AAV2i8G9**, where the galactose-binding footprint from AAV9 (Q464V, A467P, D469N, I470M, R471A, D472V, S474G, Y500F, and S501A) was engrafted into the capsids of AAV2 or **AAV2i8** (AAV2 capsid with a substitution from AAV8 to abrogate HSPG binding, reduce liver tropism, and increase muscle targeting in mice ([291])), respectively [292]. AAV2G9 maintained HSPG binding from AAV2 but gained galactose binding from AAV9, resulting in heart tropism and higher skeletal muscle, kidney, and liver transduction. AAV2i8G9 remained liver de-targeted like AAV2i8 but gained further muscle targeting, possibly due to its binding to galactose, following IV administration into mice [292]. Interestingly, point mutations in the surface-exposed tyrosine residues on the AAV2 capsid prevented capsid phosphorylation, the subsequent ubiquitination, and proteasome-mediated degradation, improving intracellular trafficking to the nucleus and enabling a 10-fold-higher HeLa cell transduction and a 30-fold-higher mouse liver transduction [293] or higher mouse retinal cell transduction after subretinal or intravitreal delivery [294]. This strategy has been adopted by other research teams and used in a phase I/II clinical trial to treat X-linked retinoschisis (NCT02416622 [200]), again highlighting the power of manipulating intracellular interactions between the AAV capsid and host cells to enhance vector performance.

To generate new capsids which transduce the CNS, the minimal footprint from AAVrh.10 that provides BBB-crossing properties was grafted into AAV1, which is unable to cross the BBB [295]. To identify the footprint, a gene-shuffling library was interrogated in mice after IV delivery for their cell type-targeting properties in the CNS and compared to AAV1 (vascular cells) and AAVrh.10 (neurons, glia, and brain endothelial cells) [119,295,296,297]. Interestingly, two variants demonstrated a more robust and selective neuronal transduction compared to AAVrh.10 and a negligible liver transduction, in contrast to AAVrh.10 or AAV1. Non-structural exposed residues originating from AAVrh.10 and differing from AAV1 were then engrafted into AAV1 to generate **AAV1RX**, a novel variant with the minimal footprint from AAVrh.10 enabling BBB crossing. This variant demonstrated broad CNS transduction in mice following IV delivery, targeting mostly neurons, some glial cells, and a few endothelial cells [119,295]. Importantly, despite the fact that CNS and heart transduction was lower than that of AAVrh.10, this variant showed more cell type specificity and substantial de-targeting from the liver, demonstrating an improved safety profile [295]. Recently, Deverman and colleagues developed a novel mechanism-focused approach based on the screening of variants through pull-down assays using known BBB receptors immobilized onto magnetic beads as Fc-fusion proteins [298]. Building upon their efforts, they identified **BI-hTFR1**, a variant which binds to the human transferrin receptor (TfR1), a protein expressed on the BBB at high levels. The authors demonstrated that BI-hTFR1 could be transported across a human brain’s endothelial cell layer in a transwell model through receptor-specific interactions, and they reported a 40-to-50-fold-increased CNS transduction in human TFRC knock-in mice compared to AAV9, with neuronal and astrocytic targeting across regions and spinal cord transduction [299]. Importantly, this novel variant only exhibited enhanced tropism for the CNS. Also, it showed interactions with only human TfR1 but not the NHP homolog, which could potentially pose a challenge to its progression to safety testing in large animal models.

In another study, Tan and colleagues engineered **AAV-ie**, which carries the BBB-crossing peptide from AAV-PHP.eB into the capsid of AAV-DJ [300]. This variant was administered through the RWM into the cochlea of infant mice and demonstrated the transduction of 75–83% of SCs across locations, outer and inner HCs, and various other cochlear cell types depending on the viral dose, in contrast to AAV-DJ. In adult mice, AAV-ie targeted the SC with lower efficiencies. Interestingly, AAV-ie was able to diffuse to the vestibular sensory organs and transduce the utricle epithelium in both infant and adult mice and showed a transduction of 93% utricular SCs and 76% HC in human vestibular epithelia harvested from an adult patient as well as the SCs and HCs from human saccule and crista.

Novel screening platforms such as BRAVE (Barcoded Rational AAV Vector evolution) have been used to engineer and identify variants using a mixed approach between rational design and directed evolution [301] (Figure 5). In the BRAVE approach, AAV2 *rep* and *cap* genes are expressed from outside the scITRs of the library construct, while the area between the scITRs includes a reporter cassette and a barcode used for variant identification during screening campaigns. In order to virtually pair the diversity region to the barcode, a small aliquot of the assembled plasmid library is incubated with Cre recombinase in vitro and, upon recombination of the LoxP sites within the construct, the area between the diversity region and the barcode is removed, bringing the two elements close in space and ready for NGS. In addition, the recovery of BRAVE variants is performed through the RNA, reducing the number of false positives. In order to engineer capsids able of retrograde transport within neurons, peptides derived from proteins known to associate to synapses were inserted into the AAV2 capsid, also leading to the disruption of the HSPG-binding motif. Upon administration to rat forebrain, the variants **MNM004** and **MNM023** displayed retrograde transport, with MNM004 reaching all afferent CNS structures [301], similarly to what had been observed with rAAV2-retro [269]. The insertion of peptides originating from canine adenovirus (CAV-2) capsid protein domains capable of retrograde transport into the AAV2 capsid resulted in **MNM008** [301], which showed improved retrograde transport from the striatum to neurons in the substantia nigra pars compacta. Retrograde transport from the frontal cortex of rats transplanted with dopaminergic neurons derived from human embryonic stem cells (hESCs) toward the neurons’ nuclei was also observed for MNM004 and MNM008, though interestingly it did not correlate with the transduction of human neurons in vitro. **MNM009** and **MNM017,** with peptides originating from a central region of the Tau protein, exhibited retrograde transport in vivo, and MNM017 also efficiently transduced primary rat neurons and astrocytes and human primary astrocytes in vitro.

The combination of different engineering strategies was also explored when an AAV2 library was engineered by replacing exposed residues within the VRs with those found via gene shuffling in silico, aligning 150 AAV serotypes, mutating known residues (Y444F and Y500F) purportedly to reduce targeting of the proteasome for degradation and of R585 and R588 for eliminating HSPG binding, and using productivity data to inform researchers regarding permissive positions [230]. From these efforts, **Li-A** and **Li-C** were selected for their high tropism for murine liver after IV delivery, which significantly exceeded that of AAV2, with Li-C reaching levels comparable to the liver-tropic AAV8.

### 4.3. Ancestral Capsid Reconstruction

Another approach to engineer AAV capsids is to computationally reconstruct ancestral AAV capsids in silico based on the DNA sequence evolution of contemporary AAV serotypes. By performing maximum-likelihood ancestral sequence reconstruction (ML-ASR), Zinn and colleagues predicted the AA sequence of putative ancestral AAV capsid monomers and identified capsid regions with evolutionary relevance that could be targeted for library mutagenesis, giving rise to **Anc80**, the predicted oldest ancestor of AAV1, 2, 8, and 9 [302]. The authors next constructed a library on Anc80 using machine learning and screening in vitro and isolated **Anc80L65**. This variant exhibited similar liver and kidney transduction to AAV8 and a moderate increase in the transduction of heart, spleen, and lung after IV delivery into mice, high muscle transduction after intramuscular injection, and high eye targeting across cell types after subretinal injection, and it transduced the mouse cochlea (inner and outer HCs) and vestibular organs when administered through the RWM [303]. In rhesus macaques after IV delivery, it showed a liver transduction superior to that of AAV8. To enhance their platform, the authors developed CombiAAV [304], a new library assembly approach based on CombiGEM [305,306] which ensures all combinations across variant positions. The authors performed a multiparametric screen, which they named AAVSeq, in mice and cynomolgus macaques and identified AA266 within Anc80 as a toggle for liver transduction (glycine enabling a 100-fold increase in mice or an 11.9-fold increase in NHP, compared to alanine). Interestingly, p3G (glycine in that position) demonstrated a shorter persistence in the serum and a lower tropism for skeletal muscle in NHP but a longer persistence in the serum in mice and lower vector genomes in the spleen in both species. Two variants from the library (**Anc80L1533** (266G) and **Anc80L1093** (266A)) were further characterized, and Anc80L1533 showed a higher tropism for mouse liver than Anc80L1093 (2622-fold in the DNA and 512-fold in the RNA). When the liver de-targeting residue was engineered into an AAV9 capsid (**AAV9-GA** and **AAV9-GAST**), it yielded 100-fold liver de-targeting in mice after IV delivery compared to AAV9 and a lower CNS, skeletal muscle, and heart transduction [304]. Finally, the mutation of this residue to glycine within the AAV3B capsid, a serotype which poorly transduces the liver, generated a novel variant (**AAV3B-AG**), with a 20-fold-enhanced liver transduction [304], in agreement with other studies [307], highlighting the ability to graft liver-targeting mutations into AAV vectors.

## 5. Mechanistic Insight for the Transduction of Novel AAV Capsid Variants

Enhancing our knowledge of the mechanisms underlaying the cellular uptake and intracellular trafficking of novel AAV variants and existing serotypes is essential to understand why they exhibit varying levels of transduction potency and efficacy across tissues and cell types. This deeper insight subsequently supports further engineering efforts aimed at fine-tuning the interactions between AAV vectors and specific host cell receptors to improve their performance. Additionally, the identification of genomic and anatomical differences between human and various animal models is pivotal for developing, characterizing, and validating novel AAV variants that are not only effective but can also successfully translate from a preclinical setting to clinical applications.

### 5.1. CNS Variants

Receptors for members of the AAV-PHP.B family have been identified. Interestingly, whilst AAV-PHP.B showed a dramatic increase in CNS tropism in C57BL/6 mice and other strains, it showed low transduction in the CNS in strains such as BALB/c mice [240,241,242]. By comparing membrane protein expression and performing whole-genome SNP and indel analysis between permissive and non-permissive mouse strains for CNS transduction as well as transcriptomic profiling of brain microcapillary endothelia, the receptor responsible for allowing AAV-PHP.B variants to cross the BBB via transcytosis was identified as glycosylphosphatidylinositol (GPI)-anchored protein lymphocyte activation protein-6A (Ly6A)/stem cell antigen-1 (Sca-1) [240,259,308,309]. This protein is located on membrane lipid rafts and expressed mainly in the endothelial cells in the CNS, albeit also in neurons and glia at lower levels [308,310], but its physiological functions are still unclear, and no ligand has been identified to date [311,312]. ELISA, surface plasmon resonance (SPR), antibody competition experiments, and the use of *Ly6a* CRISPR knocked-out BMVECs cells demonstrated that AAV-PHP.B binds to Ly6A on Ly6A-expressing cells, such as primary BMVECs, and uses it for transduction [254,259,308,309]. The assessment of the binding and transduction of Pro5 CHO cells and derivatives expressing different levels of galactose demonstrated that Ly6A serves as an attachment factor for AAV-PHP.eB independently of galactose and plays a role in the internalization and/or trafficking of virions [308]. Furthermore, Ly6A also enabled AAV-PHP.eB binding and transduction in AAVR KO mice, establishing the role of Ly6A in AAV-PHP.eB transduction in vivo and its independence from AAVR [308].

It is worth noting that the cryo-EM of AAV-PHP.B demonstrated that the inserted peptide involved in the interaction with Ly6A is highly flexible and has remarkably little impact on the surrounding capsid conformation, including the galactose-binding site [254,313], and suggested that AAV.PHP.B may bind to Ly6A through interactions involving the inserted peptide and additional residues from the VR-VIII, indicating that the peptide alone is not sufficient for binding [254]. In support of this fact, the introduction of the AAV-PHP.B peptide into AAV1 did not result in Ly6A binding and conferred a minimal transduction enhancement of Ly6A-expressing cells, and the variant failed to cross the BBB [254,313]. Furthermore, the addition of a glycine-serine-glycine linker at the N- or C-terminus flanking the peptide (**AAV1-PHP.B**) to increase flexibility did not rescue Ly6A binding, and the addition of the linker to AAV-PHP.B reduced Ly6A-mediated transduction, further indicating that the interface between the peptide and its surrounding VR-VIII residues is required for its attachment to Ly6A [254]. In agreement with this, the engraftment of a larger sequence space into AAV1 (**AAV1-PHP.B2**) enabled binding and a 20-fold increase in the transduction of Ly6A-expressing cells, similar to that observed for AAV-PHP.B. However, despite this novel variant showing a 60-fold increase in CNS transduction, it failed to penetrate the BBB and was retained in the microvascular endothelial cells. It was hypothesized that a differential affinity to Ly6A together with the differential natural receptor binding and trafficking potency between serotypes may determine the capacity of a variant to either transduce the Ly6A-expressing cells or penetrate into the CNS parenchyma via transcytosis. More recent cryo-EM studies showed that, in contrast to AAV-PHP.B, the peptide in AAV-PHP.eB exhibits a preferred spatial structure for residues D587, G588, P5* (*: position within the peptide), F6*, and K7*, with the side chains pointed toward the capsid surface [313]. On the contrary, L2*, A3*, and V4*, at a high solvent-exposed position on the outermost tip of the loop, are fully disordered and not conserved among BBB-crossing variants, suggesting that they do not form a biochemical or structural motif. Interestingly, D587 interacts with K7*, forming hydrogen bonds and electrostatic interactions holding the two sides of the loop together. This, in turn, limits the peptide’s flexibility and introduces an inward tension on the loop that results in the peptide’s tip bending downward, ultimately resulting in a favorable conformation to interact with Ly6A. This is not the case in AAV1-PHP.B, where K7* interacts with D590, pulling the peptide upward, or in AAV-PHP.B, where the lack of K7* interactions results in a highly flexible peptide and, thus, a lower binding affinity to Ly6A [313]. In addition, pull-down assays demonstrated that P5*-F6*-K7* alone are sufficient for binding to Ly6A in vitro and constitute the minimal motif for this interaction. In agreement, a newly developed integrative structure computational modeling pipeline used to model the AAV-PHP.eB–Ly6A interaction pointed at P5*-F6* as being essential for binding, showing that Ly6A interacts with one capsid monomer and that additional interactions induce steric clashes [259].

The binding of AAV-PHP.eB to AAVR has also been studied by cryo-EM. Similar to what has been found for AAV1 and AAV2, the AAVR PKD2 domain interacts with the capsid at the three-fold protrusions and the two/five-fold wall [154,313]. Most interacting residues in the capsid reside in the N-terminal, A-B loops, and B-C loops but also in strand A (E418 and I419), strand C (Y442), and strand D (I462). This is in contrast with AAV9, which uses K462 but not E418, resulting in a higher binding to AAVR. However, they show minimal differences in their capsid structure upon AAVR binding, engaging with AAVR PKD2 via VR-I, VR-III, VR-IV, VR-V, and VR-VIII, whilst AAV1 and AAV2 do not use VR-IV and VR-V [154]. In particular, residues E500 and W503 within VR-V in AAV9 showed potential contact with AAVR PKD2. Also, most variability between AAV1, 2, 9, rh.10, and AAV-PHP.eB capsid structures upon binding to AAVR occurred in VR-I, VR-II, VR-IV, and VR-VIII, with VR-I and VR-IV showing the highest diversity. Further studies showed that the bent downward angle of the peptide in AAV-PHP.eB may cause a steric clash with AAVR PKD2, explaining the weaker binding observed when it is compared with AAV9. On the other hand, the upright conformation of the peptide in AAV1-PHP.B results in its extension through the PKD2 channel, preventing clashing and, thus, allowing for binding comparable to that of the parental AAV1 [313]. In addition, PKD2 recognition was shown to be influenced more by the length of the peptide than by the size or biochemical properties of the residue side chains. Importantly, despite the fact that the binding of AAV-PHP.eB to Ly6A was reported to be stronger than that to AAVR [313], the binding of AAVR had no impact on the subsequent Ly6A binding and vice versa, indicating that distinctive sites are involved in binding to these receptors [154], in agreement with previous studies [308]. Also, a deeper characterization of these interactions using computational modeling showed that, whilst a single copy of both the Ly6A and AAVR PKD2 domains may bind to the same three-fold spike simultaneously, they achieve so without clashing [259]. A recent study adapted Retrogenix cell microarrays [314] to assess the binding of biotynilated AAVs to cells overexpressing certain receptors. Utilizing this technology, AAVR was identified as a binder for AAV.CAP-B22, AAV.CAP-Mac, AAV-MaCPNS2, and AAV9-X1.1 but not for AAV-MaCPNS1 [315].

Interestingly, whilst an ortholog with 63% homology to Ly6A is present in rats, a model which shows CNS transduction for AAV-PHP.B [243], the *Ly6A* gene in non-permissive BALB/cJ-like strains has missense SNPs (D63G or V106A) [240,308]. These mutations conform a different allelic variant called Ly6E.1 [316], with potentially different post-translational protein processing predicted to result in a lower membrane localization [308,317]. No evidence of Ly6A has been reported for other mammals such as rhesus macaques or marmosets [318], explaining why AAV-PHP.B or AAV-PHP.eB have shown limited tropism for the CNS in NHP [245,319,320,321]. Additionally, despite the fact that several genes from the LY6 family such as *LY6E* are present in the human genome [311], no direct human homolog to *Ly6A* had been found [311,318] until recently, when a close relative of murine *Ly6A* called *LY6S* was identified [322].

A recent study attempted to test the binding of existing AAV capsid variants to murine Ly6A and human LY6S and identify additional receptors used by BBB-crossing AAV variants with diverse motifs. SPR assays confirmed binding to Ly6A for AAV-PHP.eB, AAV.CAP-B10, AAV.CAP-B22, AAV-PHP.N, 9P08, and 9P16 but not for AAV9, AAV-F, AAV-PHP.C1, AAV-PHP.C2, AAV-PHP.C3, AAV-PHP.C4, 9P31, 9P36, 9P13, 9P33, and 9P39 [259]. Another study reported that AAV-PHP.V1 was able to bind to Ly6A as opposed to AAV9-X1 or AAV9-X1.1 [252]. Recently, a study confirmed the lack of binding of AAV.CAP-Mac, AAV-MaCPNS1, AAV-MaCPNS2, and AAV9-X1.1 to Ly6A [315]. To identify the receptor/s used for non-Ly6A binders, scRNA-seq was performed on CNS endothelial cells from mice, and membrane proteins with a high and specific expression in the endothelial cells were selected for further analysis [259]. Ly6C1 was validated as a receptor for AAV-PHP.N, AAV-PHP.C1, AAV-PHP.C2, AAV-PHP.C3, AAV-PHP.C4, AAV-F, AAV-PHP.eC, 9P08, 9P13, 9P33, and 9P39, enhancing transduction to different levels, as well as for AAV-Se2, which also engages with the Ly6C1 homologs Ly6C2 [323] and Ly6E for transduction [259,266]. In contrast, AAV-PHP.eB, AAV.CAP-B10, AAV.CAP-B22, 9P16, 9P31, and 9P36 were not able to use Ly6C1 for transduction [259]. Importantly, Ly6C1 is a GPI-anchored membrane protein with expression levels consistently high across inbred mouse strains [324], suggesting that Ly6C1-utilizing AAVs may be useful research tools for a variety of neuroscience studies. In addition, since AAV.CAP-B10 and AAV.CAP-B22 contain additional modifications in VR-IV which enable them to cross the BBB in marmoset, it was speculated that they may bind to other receptors in addition to Ly6A to confer them with those properties that are not present in other Ly6A binders [259]. However, no additional receptors were found when tested for binding against marmoset CNS-expressed Ly6 family members or human LY6S. The receptor for 9P31 and 9P36, variants unable to bind to Ly6A, Ly6C1, or human LY6S, was identified to be the GPI-linked enzyme primate-conserved carbonic anhydrase IV (CA-IV) [259], encoded by *CA4* in humans and *Car4* in mice and localized on the luminal surface of the brain’s endothelial cells throughout the cortex and cerebellum, where it enzymatically modulates carbon dioxide bicarbonate’s balance [325]. Interestingly, these variants did not show transduction by other membrane-associate potential receptors from the CAR family [259]. The use of predictive-binding computational models for mouse CA-IV binding to 9P31 and 9P36 variants reported that the 9P31 peptide occupies the catalytic pocket of the enzyme, where Y5* (also shared with 9P36) approaches the enzyme’s active site, and W3* is situated in an ancillary pocket. The 9P31 peptide was predicted to extend to the surface of the enzyme, where there is a considerable sequence divergence on CA-IV, preventing cross-reactivity across species and, thus, explaining 9P31 and 9P36 variant selectivity for mouse CA-IV over the human receptor. Interestingly, *Slco1c1* (also known as *Oatp1c1* and coding for a membrane anionic transporter) was able to moderately boost the transduction of cells by most AAV variants tested, suggesting that it may be a natural receptor for AAV9, driving its weak BBB-crossing properties.

The identification of receptors for other CNS tropic variants such as AAV-B1 or AAV-AS was also attempted. Cell binding assays using either Pro5 CHO cells or Lec2 CHO cells, which are SIA-deficient and have exposed galactose, showed comparable binding to both cell types, demonstrating that either both SIA and galactose have an equal contribution to AAV-B1 and AAV-AS cell attachment or that binding takes place via an unknown receptor [256,285].

On the other side, the cleavage of terminal α2,3- and α2,6-linked SIA residues in neonatal mice with neuraminidase resulted in a significantly reduced transduction of the ependymal lining of the CNS by AAV4, whilst the treatment of endoneuraminidase-N to cleave α2,8-linked polysialic acid (PSA) resulted in increased transduction in the rostral migratory stream and, to a higher extent, in the olfactory bulb [289]. In contrast, neuraminidase had no effect on AAV4.18 tropism, and endoneuraminidase-N abrogated AAV4.18 transduction in both the rostral migratory stream and olfactory bulb. These results indicate that the mutation of only three residues in AAV4 to generate AAV4.18 achieved a switch in the glycan receptor-binding specificity from α2,3-linked SIA to α2,8-linked PSA. The specific tropism of AAV4.18 for migrating progenitors in the neonatal CNS [289] can be attributed to the high levels of α2,8-linked PSA expressed on these cells [326]. In addition, the lower affinity of AAV4.18 for its receptor may allow for its higher penetration within the ependymal barrier into the CNS parenchyma, as observed following ICV injection [289].

As explained previously, striking differences in CNS tropism were observed for the SIA-binder AAV1, non-SIA-binder AAVrh.10, and their chimera AAV1RX [295]. Using structural analysis, Albright and colleagues found that the critical residues for BBB crossing in AAV1RX are adjacent to the AAV1 SIA-binding site [119]. Given the implication of residues S268, D270, N271, Y445, and G470 from AAV1 in stabilizing capsid–SIA interactions and the fact that AAV1RX did not inherit them all from AAV1, the authors suggested that the disruption of SIA binding by the mutation of any of these residues may explain, in part, the AAV1RX CNS tropic phenotype, as observed for AAVrh.10 [119,295]. Owing to the possible importance of a lack of SIA-binding as a determinant factor for CNS tropism, the authors engineered a set of mutants with varying affinities for SIA, characterized their performance in vitro and in vivo, and confirmed that AAV1RX retained the ability to bind to SIA, albeit with a lower affinity than AAV1 [119]. Since the capsids with strong SIA interactions tend to have high vascular and liver transduction but a low CNS penetration, whereas the capsids with the absence of or little SIA binding show a moderate CNS transduction efficiency, it was hypothesized that different SIA-binding affinities may correlate with the capacity of capsids to transduce the CNS after IV delivery. Indeed, they observed that SIA dependence inversely correlated with CNS entry and transduction of the CNS parenchyma, and they concluded that AAV1RX’s ability to cross the BBB is enabled by its partially attenuated SIA interactions.

A recent study used a novel technology to assess the binding of several AAV capsid variants to the human membrane’s proteome and secretome- including receptors, transporters and cytokines, to screen for yet-unidentified receptors for existing variants [315]. Low-density-lipoprotein-receptor-related-protein 6 (LRP6) was identified as a receptor for AAV9-X1.1, AAV.CAP-Mac, and AAV-BI30. LRP6 is a co-receptor of the canonical Wnt signaling pathway, which is present in various tissues [327]. Through pull-down and SPR assays, binding and functional assays on cells, as well as competition assays and modeling with AlphaFold-Multimer [328], the authors confirmed these interactions through the extracellular YWTD domains 1 and 2 (E1E2) [329] of LRP6, with variable sensitivities, and described the inserted peptide as being sufficient to drive such interactions [315]. Furthermore, glycoprotein 2 (GP2), which is specifically expressed in the pancreas [330], was identified as a receptor for AAV9-X1.1 and AAV.CAP-Mac. Interestingly, the human protein exhibited stronger effects than the mouse homolog. In addition, FAM234A, which is expressed in the CNS at low levels across various neuron types [331], was shown to bind to AAV.CAP-B22 and AAV-PHP.eB, with stronger effects observed with the mouse homolog.

Finally, BI-hTFR1 was reported to engage transferrin receptor (TfR)1 for active transport across an in vitro human BBB model via receptor-mediated transcytosis (RMT) [299]. BI-hTRF1 co-localized with markers of the early and late endosomal pathway (Rab5 and Rab7) and the trans-Golgi network (TGN46) and, at low levels, with markers of the cis-Golgi (Rcas1) or endoplasmic reticulum (KDEL), in contrast to AAV2, which only co-localized with TGN46. This is consistent with previous data describing how TfR1 clustering promotes clathrin-coated pit formation and uptake via Rab5-positive endosomes [332,333]. In turn, Rab7 decorates endosomes associated with transcytosis and the lysosomal degradation pathway [334,335]. Importantly, BI-hTFR1 was demonstrated to bind to the apical domain of TfR1, a different site than transferrin, the natural ligand for TfR1. This is crucial since variant competition for receptor binding with the natural ligand can potentially decrease vector potency or pose safety risks.

### 5.2. Skeletal Muscle and Heart Variants

Receptors for muscle and cardiac tropic AAV capsid variants have also been identified. The MyoAAV family of variants share a common RGD motif [278], which is the minimal sequence in fibronectin which facilitates binding to its receptor, the integrin heterodimer α5β1 [336]. Similarly, MyoAAV 1A was reported to use αV integrins for the transduction of mouse and human primary skeletal muscle myotubules, and demonstrated binding and transduction in vitro through αVβ6 integrins but not αVβ1, αVβ3, or αVβ8 integrins, and also to a lesser extent through α8β1 integrins [278]. MyoAAV 2A was shown to have affinity for binding to a broader class of αV integrin heterodimers, engaging with αVβ1, αVβ3, αVβ6, and αVβ8. MyoAAV 4A, 4E, 3A, and 4C exhibited the highest affinity for αVβ6, followed by αVβ8 and αVβ3. Interestingly, all the variants showed a weaker binding to αVβ6 than MyoAAV 1A. Furthermore, the role of glycan moieties on MyoAAV 1A transduction and binding were also reported. The removal of SIA from glycans to expose galactose increased binding by up to 4-fold and transduction by 172-fold in vitro, and the blockade of terminal β-1,4 galactose with *Erythrina cristagalli* lectin (ECL) significantly inhibited the binding and transduction of MyoAAV 1A. Furthermore, MyoAAV 1A, 4A, 4E, 3A, and 4C were shown to require AAVR for efficient transduction, and the overexpression of integrins only marginally rescued transduction, indicating that integrin heterodimers and AAVR play distinct roles in the entry mechanism of the MyoAAV family and, most likely, perform said roles at different stages. Regarding AAVMYO, given the presence of the RGD motif and the fact that integrin alpha-7 (ITGA7)/beta-1 is abundant in all muscle types, it was speculated that AAVMYO may interact with this integrin for its enhanced muscle transduction [276].

### 5.3. Lung and Liver Variants

AAV-QPE and AAV-VNT were found to have lost their AAV2 HSPG binding in vitro [279]. In contrast, variants from the same screening campaign that retained binding to HSPG failed to show any shift in biodistribution from AAV2, suggesting that disrupting HSPG binding may be an important factor when developing vectors with novel tropism. Regarding AAV2.5T (chimera of HSPG-binder AAV2 and SIA-binder AAV5), the use of neuraminidase on the apical side of airway epithelia significantly decreased binding, indicating the requirement of SIA. However, the use of cells with differential glycosylation demonstrated the lack of a need for HSPG [282].

Tissue specificity and the expression levels of transgene have been shown to be rather variable and unpredictable across animal models, suggesting that differential functional transduction between capsid serotypes and variants may be dependent not only on receptor engagement but also on post-uptake factors and mechanisms. A good example is AAV-LK03, a chimeric variant which shows a high transduction of human hepatocytes but no transduction of mouse hepatocytes [286] and exhibits a slower onset of transgene expression compared to AAV8. Interestingly, it was recently discovered that this variant is not defective in the mouse cell receptor binding, entry, uncoating, nuclear accumulation, or episome formation of vector genomes but rather shows a lack of histone modifications (H3K4me3 and H3K27ac) in the viral genome related to active transcription in mouse cells despite proper nucleosome assembly [192]. In contrast, the amount of repressive histone modifications (H3K9me3 and H3K27me3) is comparable to other capsid controls. As a result, AAV-LK03 genomes are eventually degraded in mouse cells since they do not form stable structures with core histones. This epigenetic regulation seems to be modulated by a single amino acid in the AAV-LK03 capsid, since its mutation restores the transduction of murine cells and is associated with the accumulation of active, related epigenetic marks. Also, the insertion of a glycine residue at position 265 in AAV-LK03 (**AAV-AM**), which extends the VR-I, allowing for a closer proximity of the adjacent alanine and serine side chains to the histidine side chain located six residue positions downstream, restored expression in mouse cells in vitro and in vivo to similar levels to those in human cells and was associated with an increase in active histone marks in the vector genomes. This study demonstrates that transgene expression from double-stranded, episomal DNA can be influenced by the nature of the AAV capsid proteins. This discovery has a profound impact on the AAV capsid engineering field for tropism modulation.

## 6. Conclusions, Perspectives, and Future Directions

Engineering and screening technologies have advanced rapidly, leading to the generation of novel variants with improved characteristics. The development of screening platforms to identify cell type-specific and transcriptionally functional AAV variants has been key in reducing the number of false positives, shortening the path toward candidate identification during screening campaigns (Figure 5). Despite the successful implementation of powerful tools, novel and improved capsid discovery platforms emerge each year. This is the case of AAVid, which leverages a massive variety of capsid libraries to generate a high-resolution map of how the AAV mutational space affects capsid assembly and tissue tropism (Shape Therapeutics, ASGCT 2023). By using this platform coupled with machine learning, the company reported the identification of CNS-targeted AAV5 capsid variants with substitutions at AA581-589 (**AAVid-C001, AAVid-C002, and AAVid-C003**) that exhibit a higher efficiency than AAV9 for the transduction of the CNS compared to the liver as well as other skeletal muscle and heart tropic variants. In addition, improvements in engineering techniques and in the acquisition and analysis of data have enabled the screening of highly complex libraries of a higher quality and produced more informative data. Although the most common peptide modifications made include a 6- or 7-mer insert/substitution, targeting peptides of up to 19 amino acids have been identified [256,301,337,338]. Despite this success, a percentage of engineered variants may exhibit productivity challenges, highly reducing the number of variants within a library which end up being dosed in preclinical studies and, thus, possibly affecting the overall success rate of screening campaigns. To overcome this, the optimization of capsid variants for productivity through generative machine learning strategies of capsid design in silico is becoming a popular strategy, as well as enriching for promising candidates for manufacture at a large scale whilst maintaining capsid stability throughout the purification and storage processes, which is critical for success in the clinic. Importantly, vector genome biodistribution can be strikingly different than transduction profiles in vivo due to variants transducing cell types unable to express the transgene or variants reaching the cell nucleus but unable to produce active transcription. A good example is AAV-LK03, whose genomes are degraded in mouse cells since they do not form stable structures with core histones [192], or AAV5, which shows high levels of vector genomes in mouse liver but a low expression of transgenes [276]. In addition, some variants such as AAV.cc47 exhibit higher transcript levels in tissues than their parental serotype, despite similar vector genome copy numbers, indicating that they may be exploiting post-entry mechanisms, accounting for their increased potency [273]. Thus, it is advised to screen for candidates through platforms enabling the recovery of transcriptionally functional variants and characterize both DNA and RNA biodistribution profiles to identify residue motifs that result in transcriptionally non-functional variants to inform engineering efforts. Together, these advances have resulted in the identification of novel AAV capsid variants that can be used for research (applications described in depth elsewhere [1]) and/or have the potential to translate into the clinical setting (Supplementary Table S1).

Despite the rapid generation of novel AAV variants in recent years, achieving tissue or cell type specificity is currently one of the main challenges in AAV gene therapy. In particular, the liver acts as a sink, capturing AAVs, resulting in a decrease in the amount of available vector for other tissues, and potentially leading to toxicity and the activation of immune responses. It has been reported that, in humans, AAV vectors administered systemically can result in transient elevation in the liver transaminase levels in patient serum, complement activation, thrombotic microangiopathy, renal failure, and, in severe cases, even death, posing safety concerns within the medical community [274,275]. De-targeting from the liver would not only increase safety but also provide the vector with more time to circulate in the blood stream to reach other tissues. Techniques such as pull-down assays, high-resolution X-ray crystallography, cryo-EM, and macromolecular modeling (e.g., Rosetta [339]) have elegantly shown that the peptides inserted into AAV capsid proteins can simultaneously enable novel receptor-binding properties and modify binding to natural receptors, serving as a tool to target and de-target variants from tissues through one single mutational strategy [230,231]. Nevertheless, cargo engineering can also help in addressing this issue by using liver-specific transcriptional regulatory elements to reduce transgene expression (e.g., miRNA-122 [340], used in AAV9-X1 [252]). In addition, lacking in most studies is the assessment of AAV variant residency in lymphatic tissues, which could provide information on humoral and cellular immune responses against variants, and the monitoring of the correlation of transduction efficiency with dose and DRG pathology, which has been observed in NHPs [341,342]. Finally, given that AAV-induced toxicity can occur within days of and up to weeks from vector administration and persist for months in both rodents and NHP, extended time points of safety and tolerability evaluations may be needed [341,343,344].

Designing AAV capsid variants able to evade capsid immune responses [345] and provide the longevity of transgene expression are also pitfalls yet to be overcome. Scientists have developed elegant strategies to inhibit or reduce the neutralization of capsids by circulating antibodies [7,214,345,346], such as by depleting anti-AAV antibodies through capsid-based apheresis [347,348] or using highly efficient bacteria-derived antibody-binding enzymes to block neutralizing antibodies (i.e. protein M) [349] or IgG-degrading enzymes (i.e., IdeS and IdeZ) to cleave human IgG [350,351,352,353]. Importantly, these approaches could be used synergistically with the engineered AAV variants and advance to the preclinical and/or clinical stage, demonstrating promising results and proven safety in NHP and/or humans. Non-invasive strategies based on the re-administration of vectors using non-cross-reactive serotypes have also been explored. The latter approach relies on the capacity to transfer targeting properties from one serotype to another and has proven successful in some cases (e.g., liver toggle into AAV9 and AAV3B [304] and brain endothelial targeting from AAV9-X1 to AAV1 and AAV-DJ [252]). Unfortunately, in most cases, the transfer of tropism across serotypes is unsuccessful or unpredictable (e.g., AAV-PHP.B peptide into AAV1 or AAV5 [253,254]), since the mutations alone may be insufficient to yield receptor-binding properties, and tropism is a multifactorial event determined by more than just capsid–receptor interactions.

Another important lesson from most of the recent studies is that AAV serotypes or variants can exhibit performance differences across species or animal strains [104]. For instance, AAV1, 5, and 6 exhibit different tropism in mice lacking the SIA-modifying CMAH enzyme, which is also lacking in humans [354]; AAV9-X1 showed different tropism and cell targeting in mouse CNS compared to its action in macaques [252]; and AAV-PHP.B and other Ly6A-binders showed BBB-crossing properties only in specific mouse strains and not in NHP [240,241,242,321]. These data indicate the lack of potential for the translatability of some BBB-crossing mechanisms from preclinical studies to a clinical setting. This could be due to anatomical differences, varying receptor expression and availability in target cells across animal models and/or disease state, differences in virus trafficking mechanisms and transcriptional activation, or differences in immune responses and binding to blood and tissue factors. Additionally, other factors such as differential spread through the tissue, sex and age, virus dose, and route of administration can also have dramatic effects on AAV performance and affect the translatability of AAV tropism, safety, and efficacy across animal models and humans. For example, the systemic injection of AAV9 leads to a higher transduction of the CNS in female mice compared to male mice [355], and a lower liver transduction is found in female specimens compared to male ones [355,356]. AAV9 transduces neurons preferentially in neonatal mice and macaques, whilst it prefers astrocytes in juvenile and adult individuals [211,239,357,358,359,360,361]. Regarding the route of administration and viral dose, it was shown that AAV9 was superior to AAV6 in transducing the myocardium when injected IV [362], but the opposite pattern was found when it was injected into the left ventricle [363]. AAV9 injected into the cisterna magna of NHP resulted in a higher DRG transduction than when injected via the lumbar cistern, and it transduced DRG at a higher efficiency after intrathecal injection [208]. Low doses (1e11 vg/mouse) of AAV-F resulted in higher enhancement of CNS transduction over AAV9 than higher doses (8e11 vg/mouse), with only higher doses resulting in CNS transduction to levels comparable to AAV-PHP.B [263]. Similarly, AAV-PHP.S showed a comparable transduction of the nodose ganglia and DRGs to that of AAV9 at low doses (3e11 vg/mouse), but the action of the former was higher at high doses (1e12 vg/mouse) [270]. As mentioned earlier, AAVs are single-stranded DNA (ssDNA) viruses that package the plus and minus strands in equal proportions. To be able to be transcribed, the ssDNA must be converted to dsDNA. It has been shown that this step can be rate limiting [175,176]. However, when the D-region and the adjacent terminal resolution site (TRS) in one of the ITRs is deleted, the replicating genome can “fold back on itself”, forming a dsDNA which is then packaged into the AAV capsid [364]. Unfortunately, while scAAVs show more rapid and higher transductions [364], the packaging capacity of scAAVs is cut in half compared to that of ssAAVs. For therapeutic applications, this can often be problematic since the length of the expression cassette, which includes the promoter, the therapeutic protein, and a polyA sequence, is, therefore, limited to roughly 2200 bp in scAAVs. Strikingly, genome configuration can also affect cell type tropism. For example, ssAAV-PHP.B targets predominantly neurons [236], while scAAV-PHP.B targets predominantly astrocytes in the CNS and spinal cord [365]. Together, the screening and characterization of novel variants may require both males and females at an age relevant to the target disease/condition, the use of several reporter constructs or the therapeutic cargo, and careful monitoring of immune responses in different animal models. Interestingly, to narrow down variants with tropism most likely to transduce into humans, several research groups have opted to perform variant screening in multiple animal models consecutively and select variants with a translatable performance [273]. Success cases include AAV.CAP-B10, AAV.CAP-B22, AAV.MaCPNS1, AAV.MaCPNS2, AAV.CAP-Mac, and AAV.cc47. Using model systems such as human-derived organoids for the screening of AAV variants could greatly accelerate engineering efforts in this field [366,367,368]. Nevertheless, despite efforts, the success rate in generating clinically relevant variants has been poor, and there is no certainty that cross-species-compatible AAV variants will be successful in showing translatable tropism in humans and clinical relevance. Thus, switching toward more rational design approaches to identify variants with binding abilities to known human receptors such as those present on the endothelial cells in the microvasculature of the CNS is a promising strategy, and several platforms are currently being characterized, validated, and incorporated into workflows. Some aspects to consider when selecting a target receptor are its expression levels across ages and sex and its uniqueness within the target cells, its biological role in a homeostasis state and in a disease, and the possible side effects resulting from engagement with the vector in question, which may depend on receptor’s kinetics for its regeneration or recycling back to the plasma membrane. Finally, assessing the pharmacokinetics of novel variants is slowly gaining popularity, since it can help scientists understand differential behaviors between variants and inform further engineering and screening campaigns.

The latest research efforts carried out at different institutions and AAV engineering companies have generated an unprecedented number of AAV capsid variants, with greater translational value given their performance across different NHP species and, in some cases, the identification of novel receptors to which they bind. These novel CNS variants include the following: 9P801, also called TTD-001 (586-AQ-587 substituted with 586-PL-587 and NGAVHLY inserted after AAV9 AA588) [369], which was screened and characterized in cynomolgus macaque, mice, and human brain microvascular endothelial cells using the TRACER platform; VCAP-101 and VCAP-102 (peptide insertion into AAV9 VR-IV), screened and characterized in adult African green monkeys, cynomolgus macaques, marmoset, and C57BL/6 and BALB/c mice (Voyager Therapeutics, ASGCT 2023); CGN2, screened in mice using the TRADE platform and characterized in cynomolgus macaque (Capsigen, ASGCT 2023); bCap 1, characterized in NHP (Dyno Therapeutics, ASGCT 2023); and a GEN5 capsid variant from Capsida Biotherapeutics, screened in adult cynomolgus macaques (ASGCT 2023). Importantly, VCAP-101, VCAP-102, bCap 1, and the Capsida GEN5 variant exhibit lower liver transduction capabilities than AAV9. Moreover, the receptor of VCAP-102 has been identified, and its binding to the human homolog has been demonstrated in vitro. Novel variants for the targeting of skeletal muscle and/or the heart in cynomolgus macaques have also been recently reported by Affinia Therapeutics (ASGCT 2023), including M1, M2, and M3. Finally, performing AAV9 engineering following a rational design approach to target an identified human receptor in the brain vasculature to achieve BBB crossing in humans, the Deverman team identified BI-huBBB1, a variant with translational potential (ASGCT 2023).

AAV capsid engineering has enabled the generation of novel variants with improved performance and a higher translational value. Proof of this is the number of engineered variants being evaluated in human clinical trials [5,117,200,370,371]. Among these, AAV-LK03 [286] has been used for the treatment of Ornithine Transcarbamylase Deficiency or Methylmalonic Acidemia in pediatric patients (NCT05092685 and NCT04581785 [200]); Spark100 or AAV-Spark200 ([372], NCT02484092, NCT03003533, and NCT03876301 [200]) have been used for hemophilia A or B; 7m8 [231] has been tested for neovascular age-related macular degeneration (NCT04645212 [200]); AAV2tYF [294] has been trialed for several eye diseases such as X-linked retinoschisis and achromatopsia ([370], NCT02599922 and NCT02935517 [200]); AAV2.5 [290] has been tested for the delivery of mini-dystrophin for Duchenne muscular dystrophy ([218,290], NCT00428935 [200]); rAAV-Oligo001 [373] has been used for Typical Canavan Disease in children (NCT04833907 [200]); 4D-R100 has been trialed for Choroideremia or retinitis pigmentosa (NCT04483440 [200]); and 4D-C102 and 4D-310 have been tested for Fabry disease (NCT04519749, NCT05629559, and NCT04519749 [200]).

Overall, the powerful capsid engineering approach has offered less invasive AAV vectors that not only expand the AAV toolbox available to researchers for studying disease and biological processes but also offer translationally relevant AAV variants that are currently being evaluated in a clinical setting. The emergence of novel platforms for molecular engineering and screening and further understanding of the mechanisms dictating the tropism of AAV vectors will enable the development of the next class of translational gene therapies with improved safety and efficacy profiles.

## Figures and Tables

**Table 1:** Primary receptors and secondary co-receptors of commonly used AAV serotypes.

SEROTYPE	RECEPTOR(S)	PUTATIVE CO-RECEPTOR(S)	REFERENCES
AAV1	α2-3 *N*-linked SIA and AAVR	Unknown	[99,107,108]
AAV2	HSPG and AAVR	FGFR1, αVβ5 integrin, α5β1 integrin, HGFR, LR, and CD9	[58,90,91,92,94,95,96,99,108,109]
AAV3	HSPG and AAVR	FGFR1, HGFR, and LR	[96,99,104,105,106,108,110,111]
AAV4	α2-3 *O*-linked SIA	Unknown	[112]
AAV5	α2-3 *N*-linked SIA and AAVR	α and β PDGFR	[99,108,112,113]
AAV6	HSPG, α2-3 and α2-6 *N*-linked SIA, and AAVR	EGFR	[99,104,105,107,114]
AAV7	Unknown	Unknown	
AAV8	LR and AAVR	Unknown	[96,99,108]
AAV9	Terminal *N*-linked galactose, and AAVR	LR and putative integrin	[96,99,108,115,116]
AAV10	Unknown	Unknown	
AAV11	Unknown	Unknown	
AAV12	Putative mannose and mannosamine	Unknown	[117]
AAV13	Unknown	HSPG	[104,105]
AAVrh.10	Sulfated *N*-acetyllactosamine (LacNAc) and AAVR	Unknown	[108,118]

SIA: sialic acid; FGFR1: fibroblast growth factor receptor 1; HGFR: hepatocyte growth factor receptor; LR: laminin receptor; EGFR: epidermal growth factor.

## Supplementary Materials

The following supporting information can be downloaded at https://www.mdpi.com/article/10.3390/v16030442/s1: Supplemental Table S1. Set of novel AAV variants discussed in this review, specifying parental serotype, mutations contained, animal species where transduction properties and/or tropism have been validated, CNS regions targeted, and receptor use switch [374,375,376].
