# Correction: Lopez-Gordo et al. Natural Adeno-Associated Virus Serotypes and Engineered Adeno-Associated Virus Capsid Variants: Tropism Differences and Mechanistic Insights. *Viruses* 2024, *16*, 442

**DOI:** 10.3390/v16091366

**Published:** 2024-08-28

**Authors:** Estrella Lopez-Gordo, Kyle Chamberlain, Jalish Mahmud Riyad, Erik Kohlbrenner, Thomas Weber

**Affiliations:** 1 Affinia Therapeutics, Waltham, MA 02453, USA; 2 Independent Researcher, South Plainfield, NJ 07080, USA; 3 Spark Therapeutics, Philadelphia, PA 19104, USA

## Update Affiliation

There was an update regarding the affiliation for Kyle Chamberlain. In the published publication, Kyle Chamberlain was as a Independent Researcher, Kyle Chamberlain most updated affiliation is affiliation **1**.

## Text Correction

In the original publication [1], AAV9-X1, AAV9-X1.1, AAV9-X1.4 and AAV9-X1.5 were erroneously referred to as AAV-PHP.X1, AAV-PHP.X1.1, AAV-PHP.X1.4 and AAV-PHP.X1.5, respectively. The X1 peptide sequence this family of variants contain (also found in AAV1-X1 and AAV-DJ-X1), AAVMYO, and AAV-Se1/Se2 were erroneously reported and have now been corrected. 

A correction has been made to Section “4.1.1. Peptide Insertion for CNS Targeting” Paragraph 5 and should read:

“In contrast to the AAV-PHP.B family, other AAV9 variants that were also recovered from CNS tissue and had motif patterns distant from it (**AAV-PHP.C1** and **AAV-PHP.C2**) showed BBB crossing across mouse strains after IV delivery [244]. AAV-PHP.C1 showed a similar astrocyte tropism and a lower neuronal targeting compared to AAV-PHP.B. AAV-PHP.C2 exhibited bias toward vascular cells and astrocytes and away from neurons, which indicates that this variant could be a good choice for non-neuronal targeting [247]. **AAV9-X1** showed the transduction of 65–70% of endothelial cells in the CNS across regions, which was higher than that for other endothelial variants, such as AAV-PHP.V1 and AAV-BR1 (variant further described below), which showed 40% transduction in this study [252]. Importantly, AAV9-X1 showed more specificity, with 95% of transduced cells being endothelial cells, compared to AAV-PHP.V1 (40% with the additional targeting of neurons and astrocytes) or AAV-BR1 (60% with the additional targeting of neurons). It is worth noting that endothelial cells from peripheral organs were not transduced by AAV9-X1. Interestingly, while AAV9-X1 efficiently transduced brain endothelial cells, liver transduction was minimal in the liver of BALB/c and CBA/J strains. To further de-target AAV9-X1 from the liver, the residue substitution from AAV-CAP.B10 was transferred to AAV9-X1, generating a novel variant called **AAV9-X1.1,** which further improved brain endothelial cell transduction across regions in mice to 82–85% and in rats. Additionally, the mutation of N272 or W503 to alanine to disrupt galactose binding resulted in two CNS endothelial variants (**AAV9-X1.4** and **AAV9-X1.5**, respectively) with reduced liver transduction. The transfer of the AAV9-X1 peptide into AAV1 (**AAV1-X1**) and AAV-DJ (variant further described below) (**AAV-DJ-X1**) resulted in improved mouse CNS transduction with endothelial cell targeting [252], indicating that the AAV9-X1 phenotype can be transferred to other serotypes, in contrast with what had previously been reported for AAV-PHP.B [253,254]. Importantly, in contrast to AAV-BR1, AAV9-X1, AAV9-X1.1, AAV1-X1, and AAV-DJ-X1 exhibited an improved transduction of human brain microvascular endothelial cells (HBMECs) over AAV9 [252]. Furthermore, AAV9-X1.1 showed an increased transduction on ex vivo brain slice cultures from southern pig-tailed macaque (interestingly, mainly neuronal) or human compared to AAV9, …”

Section “5.1. CNS Variants” Paragraph 5 and 9 should read:

“Another study reported that AAV-PHP.V1 was able to bind to Ly6A as opposed to AAV9-X1 or AAV9-X1.1 [252]. Recently, a study confirmed the lack of binding of AAV.CAP-Mac, AAV-MaCPNS1, AAV-MaCPNS2, and AAV9-X1.1 to Ly6A [315].”

“Low-density-lipoprotein-receptor-related-protein 6 (LRP6) was identified as a receptor for AAV9-X1.1, AAV.CAP-Mac, and AAV-BI30. LRP6 is a co-receptor of the canonical Wnt signaling pathway, which is present in various tissues [327]. Through pull-down and SPR assays, binding and functional assays on cells, as well as competition assays and modeling with AlphaFold-Multimer [328], the authors confirmed these interactions through the extracellular YWTD domains 1 and 2 (E1E2) [329] of LRP6, with variable sensitivities, and described the inserted peptide as being sufficient to drive such interactions [315]. Furthermore, glycoprotein 2 (GP2), which is specifically expressed in the pancreas [330], was identified as a receptor for AAV9-X1.1 and AAV.CAP-Mac. Interestingly, the human protein exhibited stronger effects than the mouse homolog. In addition, FAM234A, which is expressed in the CNS at low levels across various neuron types [331], was shown to bind to AAV.CAP-B22 and AAV-PHP.eB, with stronger effects observed with the mouse homolog.”

Section “6. Conclusions, Perspectives, and Future Directions” Paragraph 2, 3 and 4 has been corrected and should read:

“Nevertheless, cargo engineering can also help in addressing this issue by using liver-specific transcriptional regulatory elements to reduce transgene expression (e.g., miRNA-122 [340], used in AAV9-X1 [252]).”,

“The latter approach relies on the capacity to transfer targeting properties from one serotype to another and has proven successful in some cases (e.g., liver toggle into AAV9 and AAV3B [304] and brain endothelial targeting from AAV9-X1 to AAV1 and AAV-DJ [252]).”,

“AAV9-X1 showed different tropism and cell targeting in mouse CNS compared to its action in macaques [252];”.

The Supplementary Table S1 has also been updated for the same reason.

The authors state that the scientific conclusions are unaffected. This correction was approved by the Academic Editor. The original publication has also been updated.
